# The Contribution of the Predicted Sorting Platform Component HrcQ to Type III Secretion in *Xanthomonas campestris* pv. *vesicatoria* Depends on an Internal Translation Start Site

**DOI:** 10.3389/fmicb.2021.752733

**Published:** 2021-10-14

**Authors:** Christian Otten, Tanja Seifert, Jens Hausner, Daniela Büttner

**Affiliations:** Department of Genetics, Institute for Biology, Martin Luther University Halle-Wittenberg, Halle, Germany

**Keywords:** type III secretion, plant-pathogenic bacterium, *Xanthomonas*, sorting platform, internal translation initiation, C ring, chaperone, SctK proteins

## Abstract

Pathogenicity of the Gram-negative bacterium *Xanthomonas campestris* pv. *vesicatoria* depends on a type III secretion (T3S) system which translocates effector proteins into plant cells. T3S systems are conserved in plant- and animal-pathogenic bacteria and consist of at least nine structural core components, which are designated Sct (secretion and cellular translocation) in animal-pathogenic bacteria. Sct proteins are involved in the assembly of the membrane-spanning secretion apparatus which is associated with an extracellular needle structure and a cytoplasmic sorting platform. Components of the sorting platform include the ATPase SctN, its regulator SctL, and pod-like structures at the periphery of the sorting platform consisting of SctQ proteins. Members of the SctQ family form a complex with the C-terminal protein domain, SctQ_C_, which is translated as separate protein and likely acts either as a structural component of the sorting platform or as a chaperone for SctQ. The sorting platform has been intensively studied in animal-pathogenic bacteria but has not yet been visualized in plant pathogens. We previously showed that the SctQ homolog HrcQ from *X. campestris* pv. *vesicatoria* assembles into complexes which associate with the T3S system and interact with components of the ATPase complex. Here, we report the presence of an internal alternative translation start site in *hrcQ* leading to the separate synthesis of the C-terminal protein region (HrcQ_C_). The analysis of genomic *hrcQ* mutants showed that HrcQ_C_ is essential for pathogenicity and T3S. Increased expression levels of *hrcQ* or the T3S genes, however, compensated the lack of HrcQ_C_. Interaction studies and protein analyses suggest that HrcQ_C_ forms a complex with HrcQ and promotes HrcQ stability. Furthermore, HrcQ_C_ colocalizes with HrcQ as was shown by fluorescence microscopy, suggesting that it is part of the predicted cytoplasmic sorting platform. In agreement with this finding, HrcQ_C_ interacts with the inner membrane ring protein HrcD and the SctK-like linker protein HrpB4 which contributes to the docking of the HrcQ complex to the membrane-spanning T3S apparatus. Taken together, our data suggest that HrcQ_C_ acts as a chaperone for HrcQ and as a structural component of the predicted sorting platform.

## Introduction

Pathogenicity of many Gram-negative animal- and plant-pathogenic bacteria depends on the translocation of bacterial effector proteins into eukaryotic cells where they interfere with cellular processes to the benefit of the pathogen. Translocation of effector proteins is often mediated by a type III secretion (T3S) system which is a highly complex protein delivery system and structurally related to the bacterial flagellum (Büttner and Bonas, 2010; Dean, 2011; Büttner, 2016; Wagner et al., 2018). Both systems are, therefore, referred to as translocation-associated and flagellar T3S systems (Abby and Rocha, 2012). The conservation of T3S system components of flagellar and translocation-associated T3S systems from different bacterial species suggests a similar core architecture of the secretion apparatus. Conserved core components of translocation-associated T3S systems from animal-pathogenic bacteria are designated Sct (secretion and cellular translocation) followed by a letter which refers to the nomenclature of T3S system components from *Yersinia* spp. (Hueck, 1998; Deng et al., 2017; Wagner and Diepold, 2020). Structural studies revealed that several Sct proteins are involved in the assembly of the ring structures of the T3S system in the outer membrane (OM) and inner membrane (IM; Deng et al., 2017; Lara-Tejero and Galan, 2019). The OM ring of T3S systems is assembled by members of the SctC secretin family and is associated with an extracellular appendage which is referred to as T3S needle in animal- or pilus in plant-pathogenic bacteria and serves as a transport channel for secreted proteins to the host-pathogen interface (Büttner, 2012; Deng et al., 2017; Habenstein et al., 2020). The translocation of effector proteins into eukaryotic target cells is mediated by the T3S translocon, which inserts as a homo- or heterooligomeric protein channel into the eukaryotic plasma membrane (Mattei et al., 2011; Dey et al., 2019).

The IM rings of the T3S system are assembled by SctD proteins on the outer and SctJ proteins on the inner side and surround the export apparatus, which consists of an SctR_5_-SctS_4_-SctT_1_ complex situated above the IM in the periplasm as was shown for the T3S systems from *Salmonella* spp. and *Shigella flexneri* (Dietsche et al., 2016; Zilkenat et al., 2016; Kuhlen et al., 2018; Johnson et al., 2019; Miletic et al., 2021). The SctR_5_-SctS_4_-SctT_1_ complex is associated with the additional export apparatus components SctU and SctV, which insert into the IM and contain large cytoplasmic domains presumably involved in substrate binding (Büttner, 2012; Wagner et al., 2018). SctV forms a nonameric ring structure and is linked *via* members of the SctO family of coiled-coil proteins to the cytoplasmic ATPase complex of the T3S system (Gazi et al., 2008; Wagner et al., 2018; Singh and Wagner, 2019). The ATPase SctN forms a hexameric complex and is connected *via* six spoke-like structures formed by SctL dimers to six pods consisting of members of the SctQ protein family as was shown in *Salmonella* spp. and *S. flexneri* (Lara-Tejero, 2019; Lara-Tejero and Galan, 2019; Tachiyama et al., 2019). The wheel-like SctN-SctL-SctQ complex is a part of the cytoplasmic sorting platform, a dynamic structure that can assemble in the bacterial cytoplasm independently of the membrane-spanning portion of the T3S system (Diepold et al., 2015, 2017; Zhang et al., 2017; Rocha et al., 2018; Lara-Tejero, 2019; Milne-Davies et al., 2021; Wimmi et al., 2021). Recruitment of the cytoplasmic components to the IM ring of the T3S system is mediated by SctK proteins acting as linkers between the cytoplasmic domain of SctD and the SctQ pods (Diepold et al., 2010; Hu et al., 2017; Zhang et al., 2017; Tachiyama et al., 2019; Otten and Büttner, 2021).

SctQ proteins are also termed C ring proteins because their flagellar homologs FliM and FliN form a ring-like structure at the cytoplasmic side of the flagellum (Minamino et al., 2019). FliN corresponds to the C-terminal region of SctQ proteins (SctQ_C_), which are often translated as separate proteins following translation initiation at an internal start codon as shown, for example, for *Yersinia* spp., *Salmonella* spp., and *S. flexneri* (Yu et al., 2011; Bzymek et al., 2012; McDowell et al., 2016; Lara-Tejero et al., 2019). In some pathogens, however, for example, the plant pathogen *Pseudomonas syringae*, SctQ_C_ is encoded by a separate gene (Fadouloglou et al., 2004). SctQ_C_ proteins were shown to interact with SctQ and were identified as essential structural components of the T3S systems in *Yersinia* spp. and *S. flexneri* (Bzymek et al., 2012; Diepold et al., 2015; McDowell et al., 2016; Rocha et al., 2018). In *Salmonella* spp., however, SctQ_C_ was proposed to act as a chaperone which promotes the stability of SctQ (Yu et al., 2011; Lara-Tejero et al., 2019). The contribution of SctQ and SctQ_C_ to the assembly of the sorting platform and to T3S has been intensively analyzed in animal-pathogenic bacteria, whereas the exact role of corresponding HrcQ proteins from plant pathogens is still largely unknown.

In our laboratory, we study T3S in the plant-pathogenic bacterium *Xanthomonas campestris* pv. *vesicatoria* (also designated *Xanthomonas euvesicatoria*) which is the causal agent of bacterial spot disease in pepper and tomato plants (Jones et al., 2004; Timilsina et al., 2020). Pathogenicity of *X. campestris* pv. *vesicatoria* depends on the T3S system which is encoded by the chromosomal *hrp* (hypersensitive response and pathogenicity) gene cluster (Büttner and Bonas, 2010; Timilsina et al., 2020). The term *hrp* refers to the essential contribution of the gene cluster to bacterial pathogenicity in susceptible plants and the elicitation of the HR in resistant plants (Büttner and He, 2009; Deng et al., 2017). The HR is a rapid local cell death at the infection site and depends on the recognition of single type III effectors in plants with matching resistance genes (Jones and Dangl, 2006; Gill et al., 2015). *hrp* gene expression is activated when the bacteria enter the plant tissue or are cultivated in special minimal media (Schulte and Bonas, 1992). *hrp* genes are regulated by HrpG and HrpX, which are encoded outside the *hrp* gene cluster (Wengelnik et al., 1996; Wengelnik and Bonas, 1996). Eleven Hrp proteins, designated Hrc (Hrp conserved), are conserved in animal and/or plant pathogens and presumably constitute the core structural elements of the T3S system (Büttner, 2012). Among the functionally characterized Hrc proteins is the predicted C ring protein HrcQ which is encoded by the first gene of the *hrpD* operon and essential for pathogenicity and T3S (Rossier et al., 2000). We previously reported that HrcQ localizes to the bacterial cytoplasm and to the membranes under T3S-permissive conditions and that it interacts with components of the ATPase complex and the export apparatus (Lorenz et al., 2012). Fluorescence microscopy studies in *X. campestris* pv. *vesicatoria* showed that a HrcQ-sfGFP (superfolder green fluorescent protein) fusion protein forms foci in the presence of a functional T3S system, thus indicating the assembly of the predicted sorting platform (Hausner et al., 2019). Foci formation was reduced in strains lacking the non-conserved HrpB4 protein which links HrcQ to the cytoplasmic domain of the IM ring component HrcD and thus likely acts similarly to SctK proteins from animal-pathogenic bacteria (Otten and Büttner, 2021).

In the present study, we identified and analyzed a C-terminal HrcQ derivative (HrcQ_C_) which results from internal translation initiation in *hrcQ*. Complementation studies revealed that the internal translation start site is essential for pathogenicity when *hrcQ* is expressed *in cis*. Expression of *hrcQ* with mutated internal translation start site *in trans*, however, restored pathogenicity in a *hrcQ* deletion mutant overexpressing the T3S system. This suggests that the loss of HrcQ_C_ can be compensated by increased expression levels of T3S genes. Protein studies revealed that HrcQ_C_ contributes to HrcQ stability and thus likely acts as a chaperone for HrcQ. Furthermore, the results of interaction and localization studies suggest that HrcQ_C_ interacts and colocalizes with HrcQ and also binds to known HrcQ interaction partners. When analyzed by fluorescence microscopy, HrcQ_C_ colocalized with HrcQ and contributed to the assembly of HrcQ complexes. Our data, therefore, suggest that HrcQ_C_ might act as both chaperone and structural component of the predicted sorting platform.

## Materials and Methods

### Bacterial Strains and Growth Conditions

Bacterial strains and plasmids used in this study are listed in Supplementary Table S1. *Escherichia coli* strains were cultivated at 37°C in lysogeny broth (LB) medium, *X. campestris* pv. *vesicatoria* strains at 30°C in nutrient-yeast extract-glycerol (NYG) medium or minimal medium A (MA, pH 7.0) supplemented with sucrose (10mM) and casamino acids (0.3%; Daniels et al., 1984; Ausubel et al., 1996). Plasmids were introduced into *E. coli* by electroporation and into *X. campestris* pv. *vesicatoria* by electroporation or conjugation. Antibiotics were added to the media at the following final concentrations: ampicillin, 100μg/ml; kanamycin, 25μg/ml; rifampicin, 100μg/ml; spectinomycin, 100μg/ml, gentamycin, 15μg/ml; streptomycin, 25μg/ml; and nalidixic acid, 15μg/ml.

### Plant Material and Plant Infections

For infection studies, *X. campestris* pv. *vesicatoria* bacteria were resuspended in 1mM MgCl_2_ and infiltrated at densities of 1×10^8^ colony-forming units (CFU) ml^−1^ into leaves of the near-isogenic pepper cultivars Early Cal Wonder (ECW), ECW-10R and ECW-30R using a needle-less syringe (Minsavage et al., 1990; Kousik and Ritchie, 1998). Infected plants were incubated in growth chambers for 16h of light at 28°C and 8h of darkness at 22°C. The HR was documented 1–2 dpi (days post inoculation) after destaining of the leaves in 70% ethanol. Disease symptoms were photographed 1–9 dpi. The results of infection experiments were reproduced at least two times with different transformants.

### Generation of *hrcQ* Expression Constructs

For the generation of *hrcQ* expression constructs with mutations in possible start codons, construct pB-PStophrcQ containing *hrcQ* downstream of the native promoter was used as a template for PCR reactions with primers amplifying the whole plasmid and annealing to each other. Mutations of putative start codons from GTG (V1) or TTG to GCG encoding alanine were introduced by the primer sequences. The PCR amplicons were transferred into *E. coli* after a *Dpn*I digest, and constructs with single mutations were used as templates to introduce additional mutations.

To generate an expression construct encoding HrcQ-sfGFP under control of the native promoter, the annotated *hrcQ* coding sequence and 299bp upstream region were amplified from *X. campestris* pv. *vesicatoria* strain 85–10 by PCR and subcloned into pICH41021 as blunt-end fragment using *Sma*I and ligase. Subsequent ligation with *sfgfp* (construct pEX-A-sfgfp) into the *Bsa*I sites of pBRM-P by Golden Gate cloning (Engler et al., 2008) resulted in construct pB-PhrcQ-sfGFP. The mutation in codon 203 was introduced by PCR using primers which annealed back-to-back to pB-PhrcQ-sfGFP and contained *Bpi*I sites for Golden Gate-based religation of the PCR product. The M211A mutation was inserted using complementary primers annealing to pB-PhrcQ-sfGFP, and the PCR amplicon was transferred into *E. coli* after *Dpn*I digest. Using a similar strategy, mutations leading to the M203A and M211A amino acid exchanges were introduced into pB-PhrcQ which encodes HrcQ-c-Myc under control of the native promoter.

For the generation of expression constructs containing *hrcQ_M211A_*-*c-myc* downstream of the *lac* promoter, *hrcQ_M211A_* was amplified by PCR using pB-PhrcQ_M211A_ as a template. The PCR product was subcloned into pICH41021 as blunt-end fragment after *Sma*I digest and ligation. *hrcQ_M211A_* was ligated into the Golden Gate-compatible pBRM and into the *Bsa*I sites of the bacterial adenylate cyclase-based two-hybrid (BACTH) vectors pUT18_GG_, pUT18C_GG_, pKT25_GG_, and pKNT25_GG_.

For *in cis* expression of *hrcQ_M211A_* in *X. campestris* pv. *vesicatoria*, the gene was amplified by PCR using pB-PhrcQ_M211A_ as template and assembled with a module containing the native *hrcQ* promoter in pLAND-P, in frame with a C-terminal 3×c-Myc epitope-encoding sequence. To insert *hrcQ_M211A_-c-myc* into the genome of *X. campestris* pv. *vesicatoria*, pLAND-PhrcQ_M211A_ was conjugated into strains 85–10∆*hrcQ* and 85*∆*hrcQ*. Double homologous recombination events led to the insertion of *hrcQ_M211A_-c-myc* into the *hpaFG* region and were selected as described previously (Huguet et al., 1998).

To generate *hrcQ_C_* expression constructs, the corresponding region of *hrcQ* without stop codon was amplified by PCR, subcloned as blunt-end fragment into pICH41021, and ligated into the *Bsa*I sites of pBRM and the Golden Gate-compatible BACTH vectors. Additionally, *hrcQ_C_* was ligated with a PCR amplicon corresponding to the native *hrcQ* promoter into the *Bsa*I sites of pBRM-P. To generate a glutathione S-transferase (GST)-HrcQ_C_ expression construct, two modules corresponding to *hrcQ_C_* and *ptac-gst* (encodes GST under control of the *ptac* promoter) were inserted into the *Bsa*I sites of pBRM-P-stop, resulting in pB-P-ptacGST-hrcQ_C_.

### Generation of Modular T3S Gene Cluster Constructs

Modular T3S gene cluster constructs were generated as described in the Supplementary Material and are summarized in Supplementary Figure S6 and Supplementary Table S1 (Hausner et al., 2019).

### Immunodetection of Proteins

Proteins were analyzed by SDS-PAGE and immunoblotting, using antibodies directed against the c-Myc and FLAG epitope, GST, HrcJ, HrcQ, AvrBs3, and HrpB1, respectively (Knoop et al., 1991; Rossier et al., 2000). Horseradish peroxidase-labeled anti-mouse, anti-rabbit, or anti-goat antibodies were used as secondary antibodies. Binding of antibodies was visualized by enhanced chemiluminescence.

### Analysis of *in vitro* T3S

*In vitro* T3S assays were performed as described (Rossier et al., 2000). Briefly, bacteria were grown overnight in MA medium (pH 7.0) supplemented with sucrose (10mM) and casamino acids (0.3%) and resuspended in MA medium (pH 5.3) containing 50μg/ml BSA (bovine serum albumin) and 10μM thiamine at an OD_600nm_ of 0.15. The cultures were incubated on a rotary shaker overnight at 30°C, and the bacterial cells and secreted proteins were separated by filtration using low protein binding filters. Proteins in the culture supernatant were precipitated by the addition of trichloroacetic acid and resuspended in 20μl of Laemmli buffer. Total cell extracts and culture supernatants were analyzed by SDS-PAGE and immunoblotting.

### Interaction Studies Using the BACTH System

BACTH assays were performed using the EUROMEDEX BACTH system kit. Expression constructs were transformed into JM109 *E. coli* cells to analyze protein synthesis. For this, bacterial cultures were induced with IPTG (isopropyl-*β*-D-thiogalactopyranoside; 2mM final concentration) at an OD_600_ of 0.6–0.8 and incubated on a rotary shaker for 2h at 37°C. Bacterial cells were collected by centrifugation, resuspended in Laemmli buffer, and analyzed by immunoblotting, using a FLAG epitope-specific antibody.

For protein-protein interaction studies, expression constructs encoding T18 and T25 fusion proteins were cotransformed into chemically competent DHM1 or BTH101 *E. coli* strains, and transformants were plated on LB plates containing kanamycin and gentamicin. Four colonies per transformation were used to inoculate LB overnight cultures with appropriate antibiotics, which were incubated overnight at 30°C on a rotary shaker. Two μl of the overnight cultures was spotted on selective LB plates containing X-gal (5-bromo-4-chloro-3-indolyl-β-d-galactopyranoside; 40μg/ml) and 2mM IPTG. The plates were incubated at 22°C, and the color of the colonies was monitored over a period of three to 5days. The experiments were performed at least three times with four different transformants from independent cotransformations.

### GST Pull-Down Assays

For GST pull-down assays, expression constructs encoding GST, GST-HrcQ_C_, HrcQ-c-Myc, and HrcQ_M211A_-c-Myc interaction partners were introduced into *E. coli* BL21(DE3) cells and grown in LB medium until OD_600_ 0.6–0.8. Gene expression was induced by 2mM IPTG (final concentration). After 2h of incubation at 37°C, bacterial cells were harvested by centrifugation, resuspended in PBS (phosphate-buffered saline), and lysed using a French press. The lysates were centrifuged to remove cell debris, and soluble GST and GST-HrcQ_C_ proteins were immobilized on a glutathione sepharose matrix according to the manufacturer’s instructions (GE Healthcare). The matrix with immobilized GST and GST-HrcQ_C_ proteins was washed and incubated with bacterial lysates containing HrcQ-c-Myc or HrcQ_M211A_-c-Myc for 2h at 4°C on an overhead shaker. After washing of the matrix, bound proteins were eluted with Laemmli buffer. Cell lysates and eluted proteins were analyzed by SDS-PAGE and immunoblotting, using c-Myc epitope- and GST-specific antibodies.

### Fluorescence Microscopy

For fluorescence microscopy studies, *X. campestris* pv. *vesicatoria* strains were grown overnight in MA medium (pH 7.0) supplemented with sucrose (10mM) and casamino acids (0.3%). Cells were resuspended in MA medium (pH 5.3) supplemented with BSA and thiamine as described above at an OD_600nm_ of 0.15 and incubated on a tube rotator at 30°C for 1h. Bacteria were transferred onto a microscopy slide on top of a pad of 1% agarose dissolved in MA medium (pH 5.3) as described (Hausner et al., 2019). Fluorescence was inspected with a confocal laser scanning microscope (Leica STELLARIS 8) with a 60× magnification objective and 5× digital magnification. Specific filter sets were used for sfGFP (excitation at 485nm; emission at 510nm) and mKOκ (excitation at 551nm; emission at 563nm). Fluorescent foci were counted in approximately 300 cells of three transconjugants for each strain.

## Results

### A Putative Internal Translation Start Site in *hrcQ* Contributes to Protein Function and Synthesis of HrcQ_C_

**Figure 1:**
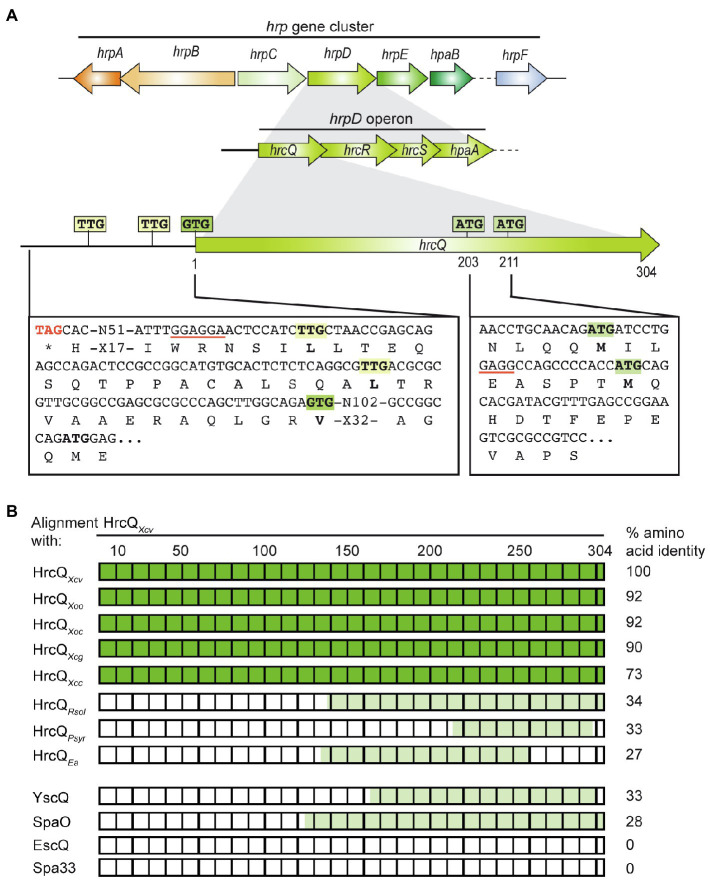
Genetic localization of *hrcQ* in the *hrp* gene cluster from *Xanthomonas campestris* pv. *vesicatoria* and alignments of HrcQ and SctQ protein sequences. **(A)** HrcQ is encoded by the first gene of the *hrpD* operon of the *hrp* gene cluster. The promoter region upstream of the annotated GTG start codon of *hrcQ* contains two TTG codons which could serve as alternative translation start sites and are located 13 and 30 codons upstream of the GTG codon. *hrcQ* also contains two potential internal translation start sites at codon positions 203 and 211. Potential Shine Dalgarno sequences are underlined. Letters refer to amino acids. Operons and genes are represented by arrows. **(B)** Comparison of HrcQ protein sequences from plant-pathogenic bacteria and SctQ proteins from animal-pathogenic bacteria. HrcQ from *X. campestris* pv. *vesicatoria* is represented by rectangles, numbers on top refer to amino acids. The percentage of amino acid identities is indicated on the right side. Protein regions of HrcQ from *X. campestris* pv. *vesicatoria* with more than 70% amino acid identities are shown in dark green and regions with lower sequence identities in light green. The following sequences were compared by pairwise sequence alignments: HrcQ from *X. campestris* pv. *vesicatoria* (*Xcv*, GenBank accession number CAJ22054), HrcQ from *X. oryzae* pv. *oryzae* (*Xoo*, GenBank accession number AAK08059), HrcQ from *X. oryzae* pv. *oryzicola* (GenBank accession number ABH07401), HrcQ from *X. citri* pv. *glycines* (*Xcg*, GenBank accession number AAP34348), HrcQ from *X. campestris* pv. *campestris* (*Xcc*, GenBank accession number CAP52441), HrcQ from *Ralstonia solanacearum* GMI1000 (*Rsol*, GenBank accession number CAD18012), HrcQ from *Pseudomonas syringae* pv. *syringae* (*Psyr*, GenBank accession number ACU65038), HrcQ from *E. amylovora* (*Ea*, GenBank accession number AAB06004), YscQ from *Yersinia enterocolitica* (GenBank accession number AAK69226), SpaO from *Salmonella enterica* subsp. *enterica* serovar *Typhimurium* (GenBank accession number AAC43863), EscQ from *Escherichia coli* O157:H7 (GenBank accession number QkQ88880), and Spa33 from *Shigella flexneri* (GenBank accession number AAP79015).

The predicted C ring component HrcQ is encoded by the first gene of the *hrpD* operon in the *hrp* gene cluster from *X. campestris* pv. *vesicatoria* and is conserved in *Xanthomonas* spp. (73–93% amino acid identity; Figure 1; Supplementary Figures S1, S2). The C-terminal region of HrcQ also shares 27–34% amino acid identity with corresponding regions of HrcQ proteins from plant pathogens and SctQ proteins from the animal pathogens *Yersinia* spp. and *Salmonella* spp. In contrast, no significant homology was detected between HrcQ and the SctQ proteins EscQ from *E. coli* and Spa33 from *S. flexneri*, suggesting that these proteins are not conserved in every species (Figure 1; Table 1).

**Table 1:** Amino acid similarities between HrcQ from *Xanthomonas campestris* pv. *vesicatoria* and HrcQ/SctQ proteins from other bacterial species.

	Percentage of amino acid identities/similarities^1^								
	HrcQ *Rsol*	HrcQ *Pss*	HrcQ_a_ *Pst*	HrcQ_b_ *Pst*	HrcQ*_Ea_*	YscQ *Yent*	Spa33 *Sflex*	SpaO *Sent*	EscQ EPEC
Length	354	393	238	137	338	307	293	303	305
HrcQ*_Xcv_*	34/48 54%	33/50 28%	41/56 11%	None	27/38^2^ 41%	33/50 46%	None	28/46 59%	None
HrcQ_C/Xcv_	40/60 84%	36/53 73%	None	30/46^2^ 77%	36/47 41%	36/61 88%	None	30/59 71%	None
HrcQ*_Rsol_*	100 100%	31/44 61%	29/41 20%	44/66 7%	None	33/56 20%	26/52 19%	26/45 48%	None
HrcQ*_Pss_*	31/44 26%	100 100%	30/47 30%	60/75 17%	30/43 96%	27/48 45%	22/39 22%	26/42 21%	None
HrcQ*_aPst_*	29/41 26%	30/47 88%	100 100%	53/63 7%	33/46 83%	44/64 14%	None	None	None
HrcQ*_bPst_*	44/66 19%	45/59 94%	50/59 16%	100 100%	53/70 43%	29/51 51%	None	25/44 59%	None
HrcQ*_Ea_*	25/40 45%	31/44 96%	33/46 58%	55/72 20%	100 100%	26/42 45%	18/40 42%	28/45 23%	None
YscQ*_Yent_*	33/56 23%	27/48 40%	44/64 12%	29/51 22%	25/40 47%	100 100%	26/44 22%	75% 28/40	None
Spa33*_Sflex_*	26/52 22%	22/39 31%	None	20/46 18%	None	26/44 23%	100 100%	24/42 98%	None
SpaO*_Sent_*	26/45 47%	26/42 27%	None	27/46 22%	28/43 21%	28/40 77%	24/47 97%	100 100%	58/75 3%
EscQ_EPEC_	None	None	None	None	None	None	None	58/75 3%	100 100%

1 The following protein sequences were used for the alignments: HrcQ from *X. campestris* pv. *vesicatoria* (*Xcv*; GenBank accession number CAJ22054), HrcQ from *R. solanacearum* (*Rsol*; GenBank accession number CAD18012.1), HrcQ from *Pseudomonas syringae* pv. *syringae* (*Pss*; GenBank accession number ACU65038.1), HrcQ from *E. amylovora* (*Ea*; GenBank accession number AAB06004.2), YscQ from *Yersinia enterocolitica* (*Yent*; GenBank accession number AAK69226.1), Spa33 from *S. flexneri* (*Sflex*; GenBank accession number AAP79015.1), SpaO from *Salmonella enterica* subsp. *enterica* serovar Typhimurium (*Sent*; GenBank accession number AAC43863.1), EscQ from enteropathogenic *E. coli* (EPEC; GenBank accession number QKQ88880.1). The percentage of query cover, that is, the percentage of the sequence which was aligned to the input sequence given on top of each column, is indicated below the percentage of amino acid identities or similarities.

2 A word size of 2 instead of 3 was used.

**Figure 2:**
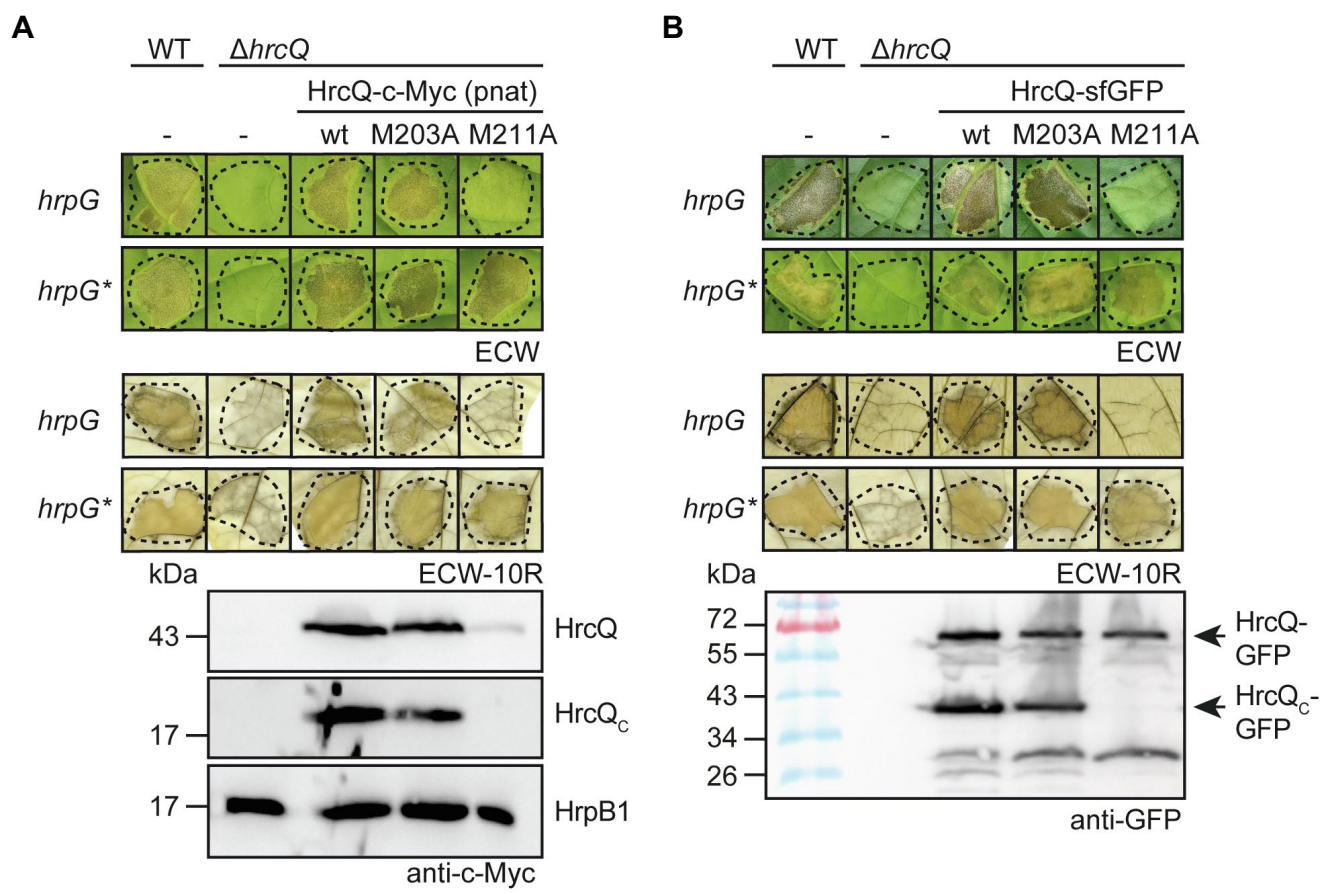
The predicted internal translation start site at codon position 211 of *hrcQ* contributes to pathogenicity of *X. campestris* pv. *vesicatoria*. **(A)** A HrcQ derivative with an M211A mutation does not restore pathogenicity in strain 85–10∆*hrcQ. Xanthomonas campestris* pv. *vesicatoria* strains 85–10 (WT, *hrpG*), 85* (WT, hrpG*) and *hrcQ* deletion mutant derivatives (∆*hrcQ*) thereof without plasmid (−) or with expression constructs encoding HrcQ-c-Myc (wt), HrcQ_M203A_-c-Myc (M203A) or HrcQ_M211A_-c-Myc (M211A) under control of the native promoter as indicated were infiltrated into leaves of susceptible Early Cal Wonder (ECW) and resistant ECW-10R pepper plants. Disease symptoms were photographed 8 dpi. For the better visualization of the HR, leaves were bleached in ethanol 2 dpi. Dashed lines indicate the infiltrated areas. For the analysis of HrcQ proteins, bacteria were grown in minimal medium and equal amounts of cell extracts were analyzed by immunoblotting using a c-Myc epitope-specific antibody. The blot shows the extracts of hrpG* strains and was reprobed with an antibody specific for the predicted inner rod protein HrpB1 to demonstrate equal loading. **(B)** The M211A mutation in a HrcQ-sfGFP fusion protein interferes with protein function. Strains 85–10, 85*, and *hrcQ* deletion mutant derivatives thereof without plasmid (−) or containing expression constructs encoding HrcQ-sfGFP fusions with or without M203A or M211A mutations were infiltrated into leaves of susceptible and resistant pepper plants. Disease symptoms and the HR were monitored as described in **(A)**. For protein detection, equal amounts of cell extracts were analyzed by immunoblotting using a GFP-specific antibody. Arrows indicate the size of HrcQ-GFP and HrcQ_C_-GFP. Experiments were performed three times with similar results.

Several *sctQ* genes from animal-pathogenic bacteria contain internal translation initiation sites which lead to the separate synthesis of the C-terminal protein regions (SctQ_C_; Yu et al., 2011; Bzymek et al., 2012; McDowell et al., 2016; Lara-Tejero et al., 2019). In *hrcQ* from *X. campestris* pv. *vesicatoria*, there are two ATG codons at positions 203 and 211 which could serve as additional internal translation start sites for the separate synthesis of the C-terminal region of HrcQ (HrcQ_C_) and are conserved in *hrcQ* genes from *Xanthomonas* spp. (Supplementary Figure S1). A potential Shine Dalgarno sequence (GAGG) is present 11 nucleotides upstream of codon 211 (Figure 1). To investigate a possible internal translation initiation in *hrcQ* transcripts, we generated expression constructs encoding HrcQ derivatives with M203A or M211A mutations (mutation of the ATG to GCG) under control of the native promoter and in fusion with a C-terminal 3×c-Myc epitope. For complementation studies, expression constructs were transferred to *X. campestris* pv. *vesicatoria hrcQ* deletion mutants, and transformants were infiltrated into leaves of susceptible and resistant pepper plants. When analyzed in *X. campestris* pv. *vesicatoria* strain 85–10∆*hrcQ*, HrcQ_M203A_-c-Myc but not HrcQ_M211A_-c-Myc restored the wild-type phenotype with respect to disease symptoms in susceptible and the HR induction in resistant plants (Figure 2A). Both HrcQ derivatives complemented the mutant phenotype of strain 85–10hrpG*∆hrcQ (85*∆*hrcQ*), which contains HrpG*, a constitutively active version of the key regulator HrpG (Figure 2A). HrpG* leads to constitutive expression of T3S genes and accelerated plant reactions (Wengelnik et al., 1999). We, therefore, assume that the negative effect of the M211A mutation was compensated by overexpression of the T3S genes in the presence of HrpG*.

Immunoblot analysis of bacterial cell extracts containing HrcQ-c-Myc or HrcQ_M203A_-c-Myc led to the detection of HrcQ and a derivative thereof at a size of approximately 20kDa corresponding to the size of the predicted internal translation product (Figure 2A). In contrast, significantly reduced amounts of the full-length protein and no internal translation product were detectable in bacterial cell extracts containing HrcQ_M211A_-c-Myc (Figure 2A), suggesting that the ATG codon at position 211 serves as additional internal translation start site and also contributes to the stability of HrcQ.

Given the low levels of HrcQ_M211A_-c-Myc, we also analyzed HrcQ-sfGFP fusions containing M203A or M211A mutations. We previously showed that HrcQ-sfGFP complements the *hrcQ* mutant phenotype and forms fluorescent foci in bacterial cells in the presence of a functional T3S system (Hausner et al., 2019). Plant infection studies showed that HrcQ-sfGFP and HrcQ_M203A_-sfGFP complemented the mutant phenotypes of strain 85–10∆*hrcQ* with respect to HR induction and disease symptoms, whereas no complementation was observed for HrcQ_M211A_-sfGFP (Figure 2B). When analyzed in strain 85*∆*hrcQ*, however, HrcQ_M211A_-sfGFP restored pathogenicity and HR induction in susceptible and resistant plants, respectively, suggesting that the negative effect of the M211A mutation was compensated by the overexpression of the T3S genes as was observed for HrcQ-c-Myc derivatives (see above; Figures 2A,B). Immunoblot analyses led to the detection of HrcQ-sfGFP and truncated derivatives thereof at sizes of approximately 30 and 40kDa, which likely correspond to cleaved sfGFP and the predicted additional translation product of HrcQ-sfGFP, respectively (Figure 2B). The M211A but not the M203A mutation abolished the detection of the truncated 40kDa HrcQ-sfGFP derivative (Figure 2B). Notably, the M211A mutation did not significantly affect the levels of full-length HrcQ-sfGFP, suggesting that the sfGFP fusion partner stabilizes HrcQ in the presence of the M211A mutation when expressed *in trans* (Figure 2B). Taken together, we conclude that the ATG codon at position 211 of *hrcQ* contributes to protein function and serves as an additional translation start site of *hrcQ*. This is supported by the presence of a potential Shine Dalgarno sequence upstream of codon 211 which is conserved in *hrcQ* genes from *Xanthomonas* spp. (Figure 1; Supplementary Figure S1).

### *In cis* Expression of *hrcQ_M211A_* Abolishes Pathogenicity

**Figure 3:**
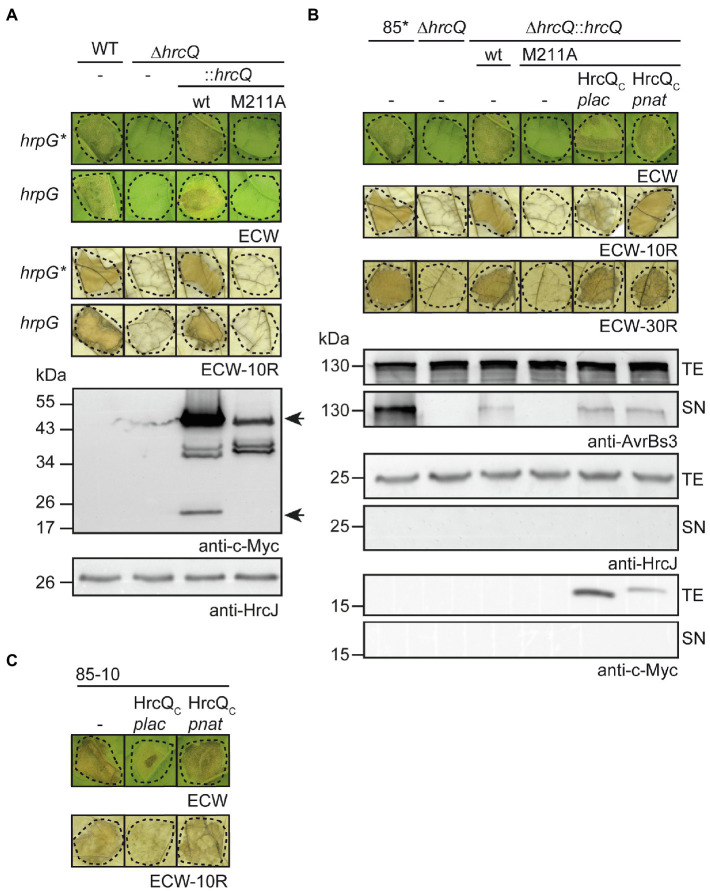
Contribution of the internal translation start codon to pathogenicity of *X. campestris* pv. *vesicatoria*. **(A)** *In cis* expression of *hrcQ_M211A_* abolishes pathogenicity. *Xanthomonas campestris* pv. *vesicatoria* strains 85–10 (WT, *hrpG*), 85* (WT, hrpG*), and *hrcQ* deletion mutant derivatives thereof containing *hrcQ-c-myc* (wt) or *hrcQ_M211A_-c-myc* (M211A) inserted into the genomic *hpaFG* region under control of the native promoter were infiltrated into leaves of susceptible ECW and resistant ECW-10R pepper plants. Disease symptoms were photographed 8 dpi. For the better visualization of the HR, leaves were bleached in ethanol 2 dpi. Dashed lines indicate the infiltrated areas. For protein analysis, bacteria were grown in minimal medium and equal amounts of cell extracts were analyzed by immunoblotting using a c-Myc epitope-specific antibody. The blot shows the extracts of hrpG* strains and was reprobed with an antibody specific for the inner membrane (IM) ring protein HrcJ to demonstrate equal loading. Arrows indicate signals corresponding to the size of HrcQ-c-Myc and the predicted internal translation product HrcQ_C_-c-Myc. **(B)** *In trans* expression of HrcQ_C_ restores pathogenicity of genomic *hrcQ_M211A_* mutants and *in vitro* type III secretion (T3S). Strains 85* and 85*∆*hrcQ* (∆*hrcQ*) with or without genomic insertion of *hrcQ-c-myc* (wt) or *hrcQ_M211A_-c-myc* (M211A) and containing either no plasmid (−) or expression constructs encoding HrcQ_C_-c-Myc under control of the native (*pnat*) or the *lac* (*plac*) promoter as indicated were infiltrated into leaves of susceptible ECW, AvrBs1-responsive ECW-10R, and AvrBs3-responsive ECW-30R pepper plants. Dashed lines indicate the infiltrated areas. Disease symptoms were photographed 8 dpi. For the better visualization of the HR, leaves were bleached in ethanol 2 dpi. For *in vitro* T3S assays, bacteria were incubated in secretion medium, and equal amounts of total cell extracts (TE) and culture supernatants (SN) were analyzed by immunoblotting using antibodies specific for AvrBs3, the IM ring component HrcJ and the c-Myc epitope. For the analysis of *in vitro* T3S of AvrBs3, *X. campestris* pv. *vesicatoria* strains contained an expression construct encoding AvrBs3 under control of the *lac* promoter. **(C)** Ectopic expression of *hrcQ_C_* under control of the *lac* promoter exerts a negative effect on pathogenicity. Strain 85–10 without plasmid (−) or containing expression constructs encoding HrcQ_C_ under control of the *lac* (*plac*) or the native (*pnat*) promoter as indicated was infiltrated into leaves of susceptible and resistant plants and plant reactions were documented as described in **(A)**. Experiments in **(A–C)** were performed three times with similar results.

Next, we investigated a possible effect of the copy number on HrcQ_M211A_ function. For this, we inserted *hrcQ_M211A_-c-myc* including the native *hrcQ* promoter into the *hpaFG* region of strains 85–10∆*hrcQ* and 85*∆*hrcQ*. The *hpaFG* region is located adjacent to the *hrp* gene cluster and serves as a landing platform for gene insertion (Tamir-Ariel et al., 2007; Lorenz et al., 2012). We previously showed that the genomic insertion of *hrcQ-c-myc* under control of the native promoter restores pathogenicity in *hrcQ* deletion mutants (Lorenz et al., 2012; Figure 3A). In contrast, expression of *hrcQ_M211A_-c-myc in cis* did not complement the phenotypes of strains 85–10∆*hrcQ* and 85*∆*hrcQ*, suggesting that the ATG codon at position 211 is essential for pathogenicity when *hrcQ* is present as a single copy in the chromosome (Figure 3A).

To analyze whether pathogenicity of genomic *hrcQ_M211A_* mutants could be restored by ectopic expression of *hrcQ_c_ in trans*, we introduced expression constructs containing *hrcQ_c_-c-myc* under control of the native or the *lac* promoter into strains 85–10*hrcQ::hrcQ_M211A_* and 85*∆*hrcQ::hrcQ_M211A_*. Immunoblot analysis of bacterial cell extracts showed that HrcQ_C_-c-Myc was stably synthesized (Figure 3B; Supplementary Figure S3; see also below). When bacteria were infiltrated into leaves of susceptible and AvrBs1-responsive resistant pepper plants, ectopic expression of *hrcQ_C_-c-myc* under control of the native promoter restored pathogenicity, whereas expression of *hrcQ_C_-c-myc* under control of the *lac* promoter only partially complemented the mutant phenotype of strain 85*∆*hrcQ::hrcQ_M211A_* but not of strain 85–10∆*hrcQ::hrcQ_M211A_* (Figure 3B; Supplementary Figure S3). Ectopic expression of *hrcQ_C_* under control of the *lac* or the native promoter in strain 85*∆*hrcQ::hrcQ_M211A_*, however, restored HR induction in ECW-30R pepper plants which recognize the effector protein AvrBs3 (Römer et al., 2009; Figure 3B). For these experiments, an *avrBs3* expression construct was additionally introduced into strain 85*∆*hrcQ::hrcQ_M211A_* and derivatives thereof. When analyzed in the wild-type strain 85–10, ectopic expression of *hrcQ_C_-c-myc* under control of the *lac* promoter interfered with pathogenicity, suggesting that it exerts a dominant-negative effect (Figure 3C). No complementation by HrcQ_C_ was observed in the *hrcQ* deletion mutant 85*∆*hrcQ*, suggesting that the N-terminal region of HrcQ is required for protein function (Supplementary Figure S3).

To investigate the effect of the internal translation start site in *hrcQ* on *in vitro* T3S, bacteria were grown in secretion medium. Total cell extracts and culture supernatants were analyzed by immunoblotting using antibodies specific for the effector protein AvrBs3 and the IM ring component HrcJ. As reported previously, deletion of *hrcQ* abolished the detectable secretion of AvrBs3 (Figure 3B; Rossier et al., 2000). T3S was restored by expression of *hrcQ-c-myc in cis*, albeit not to wild-type levels (Figure 3B). No complementation was observed upon *in cis* expression of *hrcQ_M211A_*-*c-myc*, whereas expression of *hrcQ_C_ in trans* under control of the *lac* or the native promoter partially restored T3S in strain 85*∆*hrcQ::hrcQ_M211A_* (Figure 3B). Taken together, these results suggest that HrcQ_C_ can act *in trans* and that the loss of pathogenicity of genomic *hrcQ_M211A_* mutants is, therefore, likely caused by the lack of *hrcQ_C_* expression and not by a negative effect of the M211A mutation *per se* on HrcQ stability or folding.

### Identification of Putative Translation Start Sites in the Promoter Region of *hrcQ*

**Figure 4:**
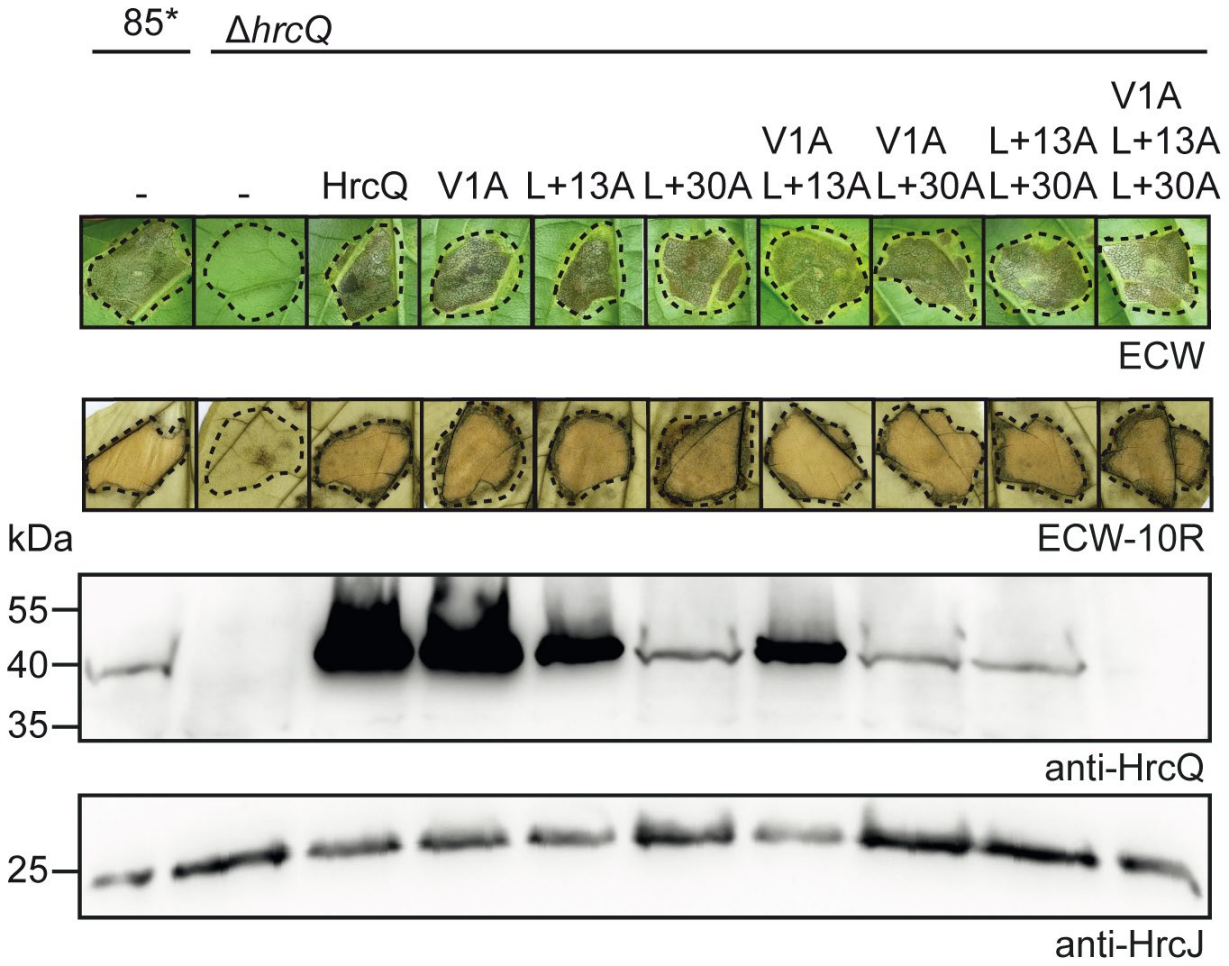
*hrcQ* derivatives with mutations in predicted start codons restore pathogenicity in a *hrcQ* deletion mutant. Strains 85* and 85*∆*hrcQ* without plasmid (−) or containing expression constructs encoding HrcQ and mutant derivatives thereof with mutations in predicted start sites as indicated under control of the native promoter were infiltrated into leaves of susceptible ECW and resistant ECW-10R pepper plants. Dashed lines indicate the infiltrated areas. Disease symptoms were photographed 8 dpi. For the better visualization of the HR, leaves were bleached in ethanol 2 dpi. For protein analysis, equal amounts of cell extracts were analyzed by immunoblotting using HrcQ- and HrcJ-specific antibodies. Experiments were performed three times with similar results.

Analysis of the additional HrcQ translation product revealed that HrcQ_C_-c-Myc migrated at a slightly higher molecular weight when encoded under control of the native instead of the *lac* promoter (Figure 3B; Supplementary Figure S3). This suggests the presence of a translation start site upstream of the annotated start codon of *hrcQ*. Inspection of the *hrcQ* promoter sequence revealed two TTG codons, 13 and 30 codons, respectively, upstream of the annotated GTG start codon, which are also present in the upstream regions of annotated *hrcQ* genes from other *Xanthomonas* spp. (Figure 1; Supplementary Figure S1). TTG codons can serve as alternative translation start sites and presumably lead to reduced gene expression (Belinky et al., 2017). Given that the translation start site of *hrcQ* has not yet been experimentally confirmed, we investigated the importance of the annotated start codon and, therefore, mutated the GTG codon to GCG in an expression construct encoding HrcQ under control of the native promoter. Complementation studies showed that the resulting HrcQ_V1A_ protein complemented the phenotype of strain 85*∆*hrcQ* with respect to disease symptoms and the HR in susceptible and resistant pepper plants (Figure 4).

To investigate a possible translation initiation upstream of the annotated start codon, we generated additional expression constructs with mutations in the TTG codons upstream of the annotated start of *hrcQ* (hereafter referred to as codon positions +13 and +30; Figure 1) leading to L-to-A (mutation of TTG to GCG) amino acid exchanges. All HrcQ derivatives containing single, double, or triple mutations complemented the mutant phenotype of strain 85*∆*hrcQ* (Figure 4). Complementation of the *hrcQ* mutant phenotype by HrcQ_V1A/L+13A/L+30A_ was confirmed in strain 85–10∆*hrcQ* (Supplementary Figure S4). Immunoblot analysis of bacterial protein extracts revealed that L+13A mutations led to reduced HrcQ levels irrespective of the presence of the annotated start codon (Figure 4). L+30A mutations further decreased HrcQ levels, whereas mutation of all three putative start codons abolished the detection of the corresponding HrcQ derivative (Figure 4). The presence of a putative Shine Dalgarno sequence eight nucleotides upstream the TTG codon at position +30, which is also conserved in the upstream regions of annotated *hrcQ* genes from other *Xanthomonas* spp. (Supplementary Figure S1) and the fact that the L+30A mutation had the most severe effect on HrcQ protein levels strongly suggests the contribution of this codon to translation initiation of *hrcQ*. Given the presence of a stop codon 72 nucleotides upstream (see Figure 1), mutation of all three potential start codons in a *hrcQ* expression construct presumably led to translation initiation downstream of the GTG codon, thus resulting in a low-level synthesis of an N-terminally truncated but functional HrcQ derivative which is not detectable by immunoblot analysis (Figure 4). An ATG codon 105 nucleotides downstream of the annotated GTG codon might serve as translation start in the construct encoding HrcQ_V1A/L+13A/L+30A_ (Figure 1). Taken together, we conclude that translation of the native *hrcQ* gene is initiated at the TTG codon located 30 codons upstream of the annotated GTG start site. Notably, the annotated start site of *hrcQ* from *X. campestris* pv. *campestris* is located immediately upstream of the TTG codon which is conserved in *hrcQ* and upstream sequences of *Xanthomonas* spp. (Supplementary Figure S1).

### Interaction Studies With HrcQ_C_

To investigate whether HrcQ and HrcQ_C_ interact with each other as reported for SctQ and SctQ_C_ proteins from animal-pathogenic bacteria (Diepold, 2020), we used a BACTH assay, which depends on the reconstitution of the catalytic domain of the adenylate cyclase (Cya) from two subdomains (T18 and T25). The interaction of T18 and T25 fusion proteins leads to the assembly of functional Cya and thus to the synthesis of cAMP, which activates the expression of the *lac* operon in *E. coli* reporter strains lacking the native *cya* gene (Karimova et al., 1998; Battesti and Bouveret, 2012).

**Figure 5:**
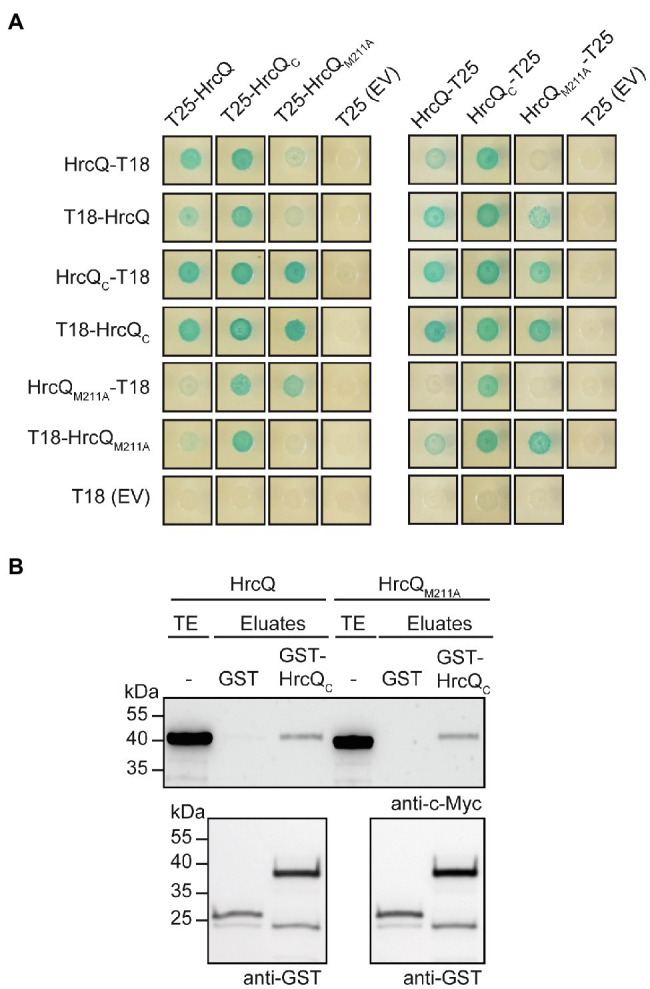
Interaction studies with HrcQ and HrcQ_C_. **(A)** *In vivo* interaction studies with HrcQ derivatives using the BACTH system. Expression constructs encoding T18 and T25 fusions of HrcQ, HrcQ_M211A_ and HrcQ_C_ were cotransformed into *E. coli* DHM1 cells as indicated. As controls, T18 and T25 fusions were cotransformed with the corresponding empty vectors (EV) encoding T18 or T25. Bacterial cultures were grown on indicator plates and photographs were taken after 3days. Spots of one representative culture per interaction are shown. Cotransformations were performed three times. Four different transformants for every combination were analyzed in every experiment with similar results. All proteins were stably synthesized as is shown in Supplementary Figure S5. **(B)** *In vitro* interaction studies with HrcQ and HrcQ_C_. GST and GST-HrcQ_C_ were immobilized on glutathione sepharose and incubated with bacterial lysates containing HrcQ-c-Myc (HrcQ) or HrcQ_M211A_-c-Myc (HrcQ_M211A_) as indicated. Total cell extracts (TE) and eluted proteins (eluates) were analyzed by immunoblotting using c-Myc epitope- and GST-specific antibodies. Experiments were performed three times with similar results.

For BACTH assays, we generated expression constructs encoding HrcQ, HrcQ_C,_ and HrcQ_M211A_ as N- or C-terminal fusion partners of the T18 and T25 domains. Immunoblot analysis of bacterial cell extracts revealed that all HrcQ fusion proteins were stably synthesized (Supplementary Figure S5). Protein-protein interactions were analyzed in the *E. coli* reporter strain DHM1, and bacteria were grown on indicator plates containing X-Gal and IPTG. We detected an interaction of HrcQ with itself and with HrcQ_C_ in all possible combinations (Figure 5A). Given that the self-interaction of the full-length protein was detected with N-terminal T18 and T25 fusion partners, it likely did not result from the interaction of two HrcQ_C_ derivatives. When compared with the HrcQ self-interaction, the interaction of HrcQ with HrcQ_M211A_ and the self-interaction of HrcQ_M211A_ appeared to be reduced for several combinations (Figure 5A). To confirm the results of the BACTH studies, we performed *in vitro* GST pull-down assays. For this, GST and GST-HrcQ_C_ were immobilized on a glutathione sepharose matrix and incubated with bacterial lysates containing C-terminally 3×c-Myc epitope-tagged HrcQ and HrcQ_M211A_, respectively. HrcQ-c-Myc and HrcQ_M211A_-c-Myc coeluted with GST-HrcQ_C_ but not with GST, which confirms the interaction between the full-length HrcQ protein and HrcQ_C_ (Figure 5B).

**Figure 6:**
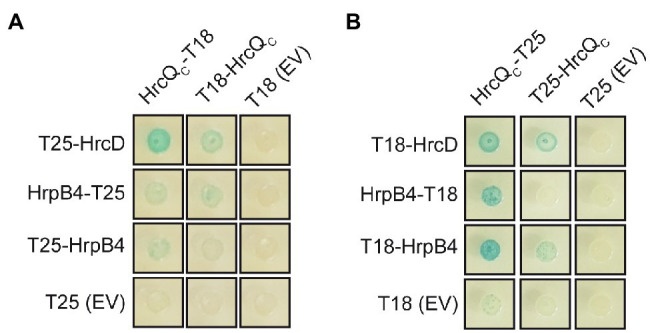
HrcQ_C_ interacts with HrcD and HrpB4. **(A,B)** HrcQ_C_ interacts with HrcD and HrpB4. T25 and T18 fusions of HrcQ_C_, HrpB4, and HrcD were cotransformed into DHM1 cells as indicated. As controls, T18 and T25 fusions were cotransformed with the corresponding empty vectors (EV) encoding T18 or T25. Bacterial cultures were grown on indicator plates and photographs were taken after 3days. Spots of one representative culture per interaction are shown. All fusion proteins were stably synthesized as shown previously (Otten and Büttner, 2021). Cotransformations were performed three times. Four different transformants for every combination were analyzed in every experiment with similar results.

We also investigated whether HrcQ_C_ interacts with the IM ring component HrcD and the cytoplasmic HrpB4 protein. We previously identified HrcD and HrpB4 as interaction partners of HrcQ and reported that HrpB4 likely acts similarly to SctK proteins from animal pathogens as a linker between HrcQ and the cytoplasmic domain of HrcD (Otten and Büttner, 2021). In the present study, the results of BACTH assays showed that HrcQ_C_ interacts with both HrcD and HrpB4 when analyzed as T18 or T25 fusion, suggesting a possible contribution of HrcQ_C_ to the docking of HrcQ complexes to the IM ring (Figures 6A,B).

### Localization Studies With Fluorescent HrcQ and HrcQ_C_ Fusion Proteins

**Figure 7:**
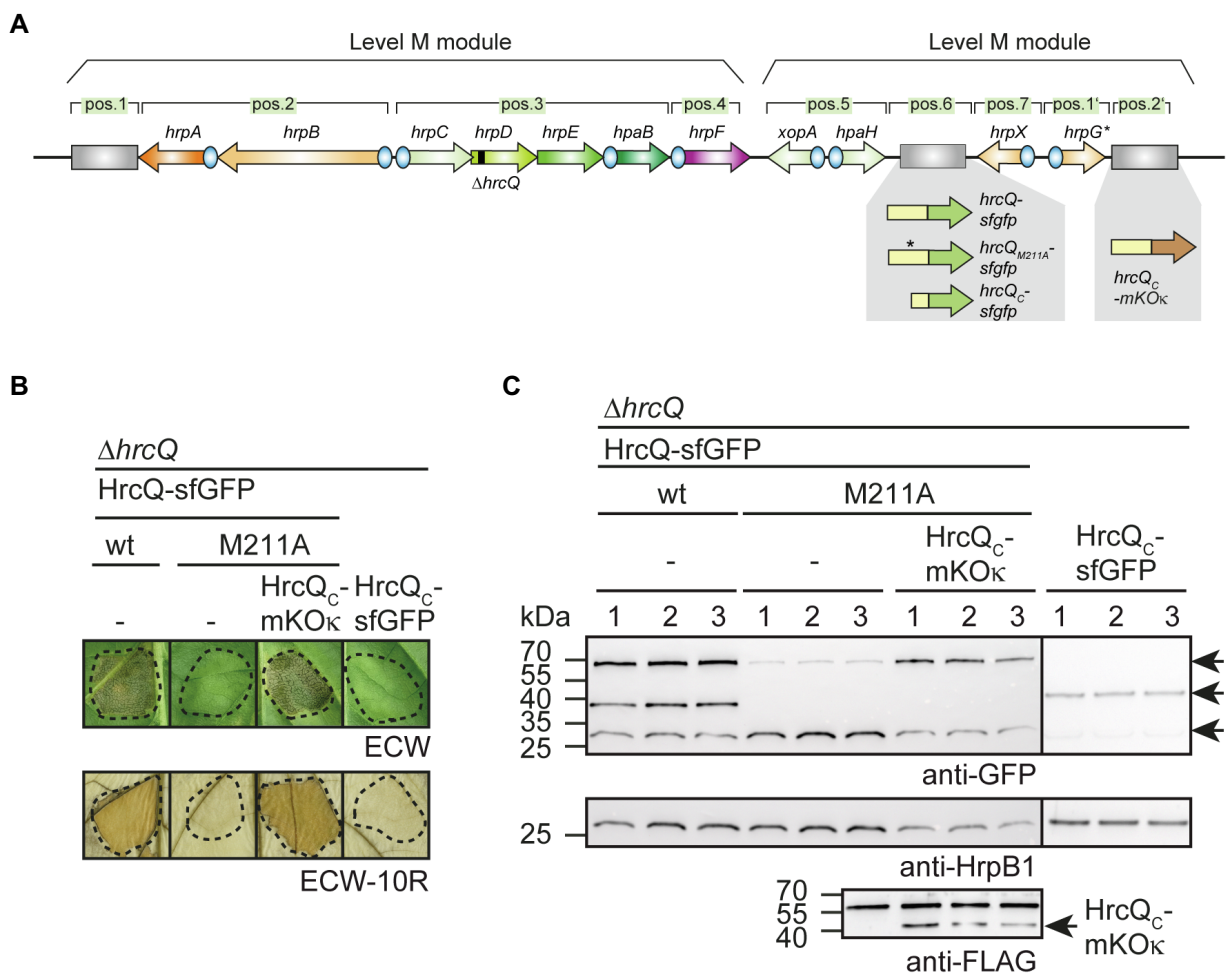
Analysis of fluorescent HrcQ and HrcQ_C_ fusions. **(A)** Schematic representation of the modular T3S gene cluster construct. Genes are represented by arrows, promoters by blue circles. Grey rectangles represent dummy modules that were replaced by reporter fusions such as *hrcQ-sfgfp*, *hrcQ_M211A_-sfgfp*, and *hrcQ_C_-mKOκ* as indicated. The deletion in *hrcQ* is represented by a black rectangle. The names of single operons and genes are given above the arrows. The constructs were assembled in different steps using the Golden Gate-based modular cloning technique as described previously (Hausner et al., 2019). Specific overhangs of gene or operon modules determined their positions (pos.) in the final level P constructs which were assembled from two level M modules as indicated. **(B)** Complementation studies with fluorescent HrcQ fusions. Strain 85*∆*hrp_*fs*HAGX* with plasmids containing the modular T3S gene cluster with a deletion in the native *hrcQ* gene (∆*hrcQ*) and including accessory and regulatory genes as depicted in **(A)** was used for the experiments. The modular T3S gene cluster constructs encoded HrcQ-sfGFP (wt), HrcQ_M211A_-sfGFP (M211A) or HrcQ_C_-sfGFP at position 6 and HrcQ_C_-mKOκ at position 2' of the modular construct (depicted in **A**) as indicated and bacteria were infiltrated into leaves of susceptible ECW and resistant ECW-10R pepper plants. Dashed lines indicate the infiltrated areas. Disease symptoms were photographed 8 dpi. For the better visualization of the HR, leaves were bleached in ethanol 2 dpi. **(C)** Immunological detection of fluorescent HrcQ fusions. Three transconjugants (labeled 1, 2, and 3) of each strain described in **(B)** were cultivated in minimal medium (T3S-permissive conditions), and cell extracts were analyzed by immunoblotting using antibodies specific for GFP and HrpB1. HrcQ_C_-mKOκ contains a C-terminal FLAG epitope and was detected using a FLAG-specific antibody. The signals corresponding to the size of HrcQ-sfGFP, HrcQ_C_-sfGFP, and a GFP cleavage product are indicated by arrows in the upper blot. The arrow in the lower blot indicates the signal corresponding to the size of HrcQ_C_-mKOκ. The additional signal at the size of approximately 60kDa presumably results from unspecific binding of the antibody and is also detected in protein extracts which do not contain FLAG epitope-tagged proteins. Experiments were performed three times with similar results.

Next, we analyzed the contribution of the internal translation start site on complex formation by a HrcQ-sfGFP fusion protein. Previous fluorescence microscopy studies showed that a HrcQ-sfGFP reporter fusion assembles into complexes (Hausner et al., 2019). For these experiments, we used a modular expression construct containing the *hrp* gene cluster, regulatory (hrpG* and *hrpX*) and accessory (*hpaH* and *xopA*) genes (Hausner et al., 2019). The accessory genes encoding HpaH and XopA contribute to the assembly of the T3S system in the periplasm and to efficient effector translocation, respectively (Noël et al., 2002; Hausner et al., 2017). The modular design of the construct, which is referred to as *hrp_HAGX* and was assembled by Golden Gate cloning from single gene and promoter modules, allows the insertion of reporter genes and the deletion of single or multiple genes of the T3S gene cluster (Hausner et al., 2019). *hrcQ-sfgfp* was inserted at position 6 of a modular T3S gene cluster lacking the native *hrcQ* gene (Figure 7A; Supplementary Figure S6). The resulting construct was analyzed in the *hrp*-deficient *X. campestris* pv. *vesicatoria* strain 85*∆*hrp_*fs*HAGX*, which lacks the T3S gene cluster and functional *hrpG*, *hrpX*, *xopA*, and *hpaH* genes. As reported previously, the modular construct restored pathogenicity in strain 85*∆*hrp_*fs*HAGX*, suggesting that it was functional and that HrcQ-sfGFP complemented the *hrcQ* mutant phenotype (Hausner et al., 2019; Figure 7B).

To analyze the influence of the internal translation start site in *hrcQ-sfgfp*, we generated modular T3S gene cluster constructs lacking the native *hrcQ* gene and containing *hrcQ_M211A_-sfgfp*, *hrcQ_C_-sfgfp* or a combination of *hrcQ_M211A_-sfgfp* and *hrcQ_C_-mKOκ* (mKusabira Orange kappa; Tsutsui et al., 2008). mKOκ is an orange fluorescent protein, used here for colocalization of HrcQ and HrcQ_C_. *sfgfp* and *mKOκ* fusions were inserted adjacent to the *hrp* gene clusters at positions 6 and 2′ of the modular constructs (Figure 7A). Plant infection experiments revealed that the *hrcQ* mutant phenotype was complemented by HrcQ-sfGFP but not by HrcQ_M211A_-sfGFP with respect to disease symptoms and the HR (Figure 7B). This is in line with the results obtained for the genomic *hrcQ_M211A_* mutation and confirms the finding that the M211A mutation interferes with HrcQ function (see above). Coexpression of HrcQ_M211A_-sfGFP with HrcQ_C_-mKOκ restored pathogenicity in the absence of the native *hrcQ* gene, suggesting that HrcQ_C_ can act *in trans* as described above (Figure 7B). Immunoblot analysis revealed that all proteins were stably synthesized (Figure 7C). The M211A mutation abolished the detection of the HrcQ_C_-sfGFP derivative and led to reduced levels of the full-length HrcQ-sfGFP protein (Figure 7C). Notably, wild-type protein levels of HrcQ_M211A_-sfGFP were restored in the presence of HrcQ_C_-mKOκ, suggesting that reduced stability was not caused by the M211A mutation *per se* (Figure 7C).

**Figure 8:**
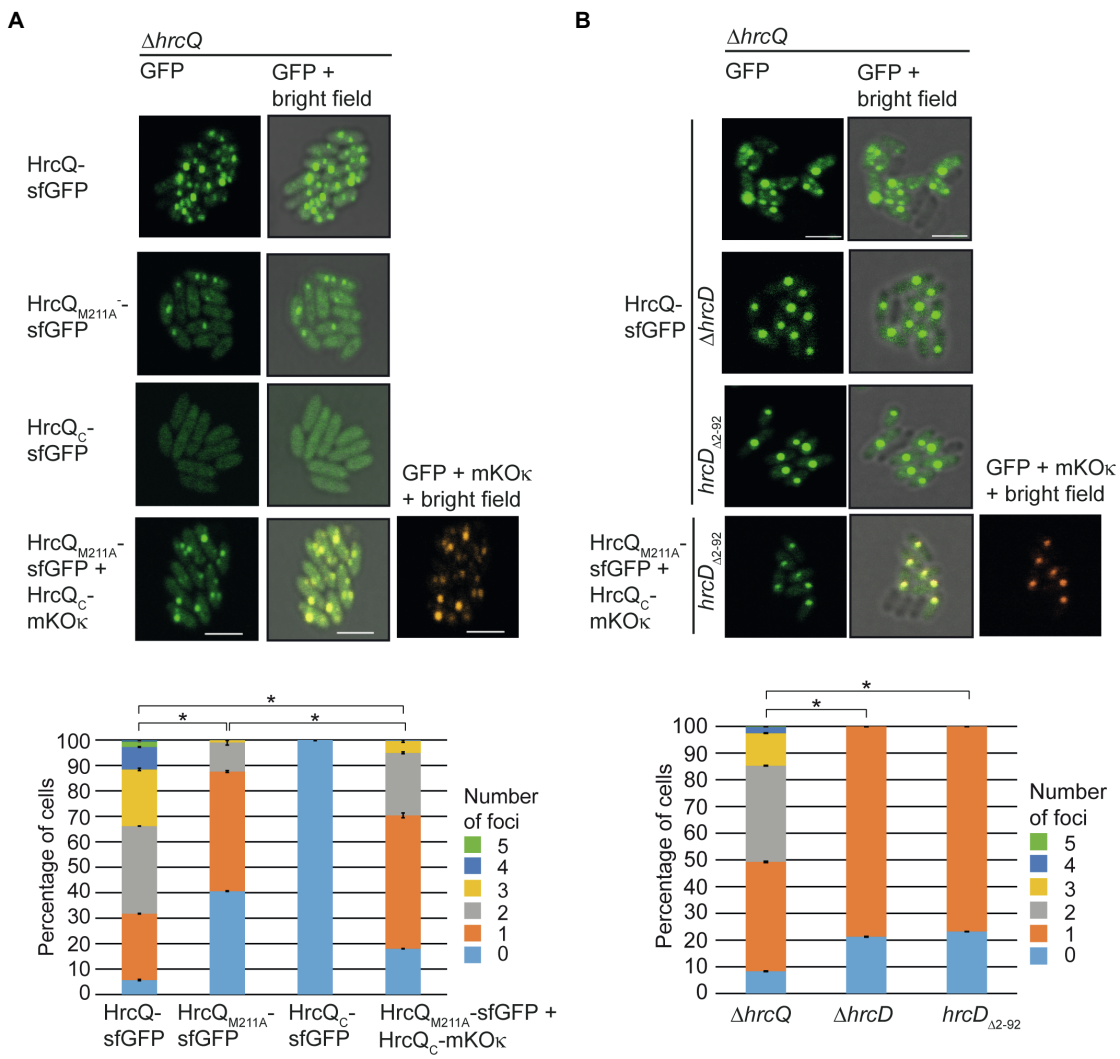
Fluorescent fusions of HrcQ and HrcQ_C_ colocalize independently of their association with the T3S system. **(A)** Colocalization of sfGFP fusions of HrcQ_M211A_ and HrcQ_C_. *Xanthomonas campestris* pv. *vesicatoria* strain 85*∆*hrp_*fs*HAGX* with plasmids containing the modular T3S gene cluster, accessory, and regulatory genes with a deletion in the native *hrcQ* gene (∆*hrcQ*) and encoding HrcQ-sfGFP, HrcQ_M211A_-sfGFP, HrcQ_C_-sfGFP, or a combination of HrcQ_C_-sfGFP and HrcQ_C_-mKOκ as indicated were incubated under T3S-permissive conditions and analyzed by fluorescent microscopy. One representative image for every strain is shown. The size bar corresponds to 2μm. The pictures in the right panels result from an overlay of the signals from the fluorescent channel for GFP and/or mKOκ with the bright field images. Fluorescent foci were counted in approximately 300 cells per strain in three transconjugants. Asterisks indicate a significant difference between the number of foci in strains with a *p*<0.05 based on the results of a chi-squared test. Experiments were performed three times with similar results with different transconjugants for each strain. The results from one representative experiment are shown. **(B)** Fluorescent fusions of HrcQ_M211A_ and HrcQ_C_ colocalize in the bacterial cytoplasm. Strain 85*∆*hrp_*fs*HAGX* with plasmids containing the modular T3S gene cluster, accessory, and regulatory genes with a deletion in the native *hrcQ* gene (∆*hrcQ*), additional deletions in *hrcD* (∆*hrcD*), or codons 2–92 of *hrcD* (*hrcD_∆2–92_*) and encoding HrcQ fusion proteins as indicated was grown in secretion medium. The formation of fluorescent foci was analyzed as described in **(A)**. Experiments were performed three times with similar results.

For localization studies, *X. campestris* pv. *vesicatoria* bacteria were grown under T3S-permissive conditions in minimal medium (pH 5.3) and inspected with a confocal laser scanning microscope. As observed previously, HrcQ-sfGFP formed one to four fluorescent foci per cell (Figure 8A; Hausner et al., 2019; Otten and Büttner, 2021). No foci were detected when the *hrpA-hpaB* operons had been replaced by dummy modules, confirming that the assembly of HrcQ-sfGFP depends on the T3S system (Hausner et al., 2019). The presence of the M211A mutation reduced foci formation by HrcQ-sfGFP (Figure 8A). HrcQ_C_-sfGFP itself did not form foci in the absence of the full-length HrcQ protein (Figure 8A). The analysis of sfGFP and mKOκ fluorescence revealed that HrcQ_M211A_-sfGFP and HrcQ_C_-mKOκ colocalize, suggesting that HrcQ and HrcQ_C_ are part of the same protein complex (Figure 8A). We, therefore, assume that HrcQ_C_ is a component of the predicted sorting platform and promotes complex formation by HrcQ.

### HrcQ_C_ Is Part of the Predicted Sorting Platform Which Associates With the Cytoplasmic Domain of the IM Ring Component HrcD

We previously showed that efficient foci formation by HrcQ-sfGFP in *X. campestris* pv. *vesicatoria* depends on the IM ring component HrcD. Thus, in the absence of HrcD, HrcQ-sfGFP forms one bright fluorescent spot which presumably corresponds to a cytoplasmic HrcQ-containing protein complex (Otten and Büttner, 2021). HrcQ is likely attached *via* the SctK-like protein HrpB4 to the cytoplasmic N-terminal domain of the IM ring component HrcD (Otten and Büttner, 2021). To confirm this hypothesis, we deleted codons 2–92 of *hrcD* in the modular T3S gene cluster construct and analyzed the effect of this mutation on bacterial pathogenicity and on the localization of HrcQ-sfGFP, HrcQ_M211A_-sfGFP and HrcQ_C_-mKOκ.

As expected, deletion of the N-terminal domain of HrcD led to a loss of pathogenicity of strain 85*∆*hrp_*fs*HAGX* containing corresponding modular T3S gene cluster expression constructs but did not affect the stability of HrcQ derivatives (Supplementary Figure S7). Fluorescence microscopy studies showed that HrcQ-sfGFP forms one bright fluorescent spot in *hrcD* deletion and *hrcD_∆2–92_* mutant strains, suggesting that the cytoplasmic domain of HrcD is required for efficient foci formation by HrcQ-sfGFP (Figure 8B). To investigate whether HrcQ_C_ is part of the cytoplasmic protein complex, we performed colocalization studies as described above with HrcQ_M211A_-sfGFP and HrcQ_C_-mKOκ. Both proteins were stably synthesized and colocalized in the absence of the cytoplasmic domain of HrcD in one fluorescent spot per cell (Figure 8B; Supplementary Figure S7). This is in agreement with the hypothesis that HrcQ_C_ associates with HrcQ-containing protein complexes and shows that the colocalization of HrcQ and HrcQ_C_ does not depend on the docking of the HrcQ complex to the cytoplasmic domain of HrcD.

## Discussion

HrcQ from *X. campestris* pv. *vesicatoria* is a conserved Hrc component of the T3S system and likely involved in the assembly of the predicted cytoplasmic sorting platform. Comparative sequence analyses revealed that HrcQ shares limited sequence similarities with other HrcQ proteins and with SctQ proteins from animal-pathogenic bacteria. Notably, no similarity was detected between HrcQ and the SctQ proteins EscQ and Spa33 from EPEC and *S. flexneri*, respectively, suggesting that HrcQ and SctQ proteins are not conserved in all species (Figure 1). We also noticed that the N-terminal regions of HrcQ proteins from plant pathogens are highly sequence variable and differ in length. This is presumably due to different positions of the annotated translation start codons, which have not been experimentally validated in most cases (Supplementary Figure S2). Our findings suggest that translation of *hrcQ* from *X. campestris* pv. *vesicatoria* is initiated upstream of the annotated GTG start codon, thus resulting in a protein with 30 additional amino acids. Unexpectedly, the analysis of HrcQ derivatives with mutations in all three potential start codons revealed that the N-terminal region is dispensable for the contribution of HrcQ to pathogenicity. The finding that the N-terminal HrcQ deletion derivative was not detectable by immunoblot analysis suggests that low levels of HrcQ are sufficient to promote T3S.

We also investigated the presence of an additional internal translation start codon which was reported for the *sctQ* genes *spaO* and *ssaQ* in the SPI-1 and SPI-2 T3S gene clusters from *Salmonella* spp. (Yu et al., 2011; Bernal et al., 2019; Lara-Tejero et al., 2019) as well as for *yscQ* from *Yersinia* spp. (Bzymek et al., 2012; Diepold et al., 2015) and *spa33* from *S. flexneri* (McDowell et al., 2016; Kadari et al., 2019). The analysis of mutant HrcQ derivatives showed that the ATG codon at position 211 is required for the detection of the additional *hrcQ* translation product, suggesting that this codon serves as internal translation start site leading to the synthesis of HrcQ_C_ (Figure 2). Notably, complementation studies revealed that expression of *hrcQ_M211A_ in trans* restored pathogenicity in *hrcQ* deletion mutants when analyzed in strains which constitutively expressed the T3S genes in the presence of HrpG*, a mutant derivative of the key regulator HrpG. In contrast, no complementation was observed in *hrpG* wild-type strains and when *hrcQ_M211A_* was expressed either *in cis* as a single copy gene or coexpressed with the T3S genes on the same plasmid (Figures 2, 3, 7). This suggests that the loss of internal translation initiation can be compensated by increased expression levels of HrcQ_M211A_ and T3S system components and points to a role of HrcQ_C_ as accessory rather than essential structural component of the T3S system.

In animal-pathogenic bacteria, truncated translation products of *sctQ* genes likely act as accessory proteins or essential structural components of the T3S system. Mutations of the internal translation start sites in *spaO* and *ssaQ* encoded in the SPI-1 and SPI-2 clusters from *Salmonella* spp., respectively, did not abolish T3S, suggesting that SpaO_C_ and SsaQ_C_ are not essential for the activity of the T3S system (Yu et al., 2011; Bernal et al., 2019). Notably, however, low levels of SpaO_C_, which presumably resulted from proteolysis, were still produced when the internal start codon was mutated and could be sufficient to restore T3S (Bernal et al., 2019; Lara-Tejero et al., 2019). In contrast to the findings reported for SpaO and SsaQ, mutations of the internal translation start sites in *yscQ* from *Yersinia* spp. and *spa33* from *S. flexneri* abrogated T3S (Bzymek et al., 2012; McDowell et al., 2016). This points to an essential role of YscQ_C_ and Spa33_C_ in T3S. It should, however, be noted that mutations in the internal start codons of *sctQ* genes from *Salmonella* spp., *Yersinia* spp., and *S. flexneri* were introduced into the genome by allelic exchange (Bzymek et al., 2012; Diepold et al., 2015; McDowell et al., 2016; Lara-Tejero et al., 2019). It would be interesting to investigate whether enhanced expression of T3S genes in combination with expression of *sctQ* mutant derivatives *in trans* could restore T3S in *Yersinia* spp. and *S. flexneri sctQ_C_* mutant strains as observed in *X. campestris* pv. vesicatoria hrpG* strains upon expression of *hrcQ_M211A_ in trans*.

In the present study, immunoblot analysis of protein extracts revealed that the M211A mutation led to decreased HrcQ levels, suggesting that HrcQ_C_ stabilizes the full-length HrcQ protein (Figures 2, 3). The effect of the M211A mutation on protein function and HrcQ stability was likely due to the absence of internal translation initiation and not caused by an effect of the mutation *per se* on protein folding or stability because pathogenicity in *hrcQ_M211A_* mutant strains was restored upon expression of *hrcQ_C_ in trans* (Figure 3). Furthermore, wild-type levels of HrcQ_M211A_ derivatives were restored upon separate expression of *hrcQ_C_*, suggesting that HrcQ_C_ stabilizes the full-length HrcQ protein (Figure 7). These findings demonstrate that HrcQ_C_ is functional when encoded by a separate gene and might act as a chaperone for the full-length HrcQ protein as was proposed for SpaO_C_ and SsaQ_C_ from *Salmonella* which stabilize the respective full-length proteins (Yu et al., 2011; Bernal et al., 2019; Lara-Tejero et al., 2019). A predicted role of HrcQ_C_ as a chaperone for HrcQ is in line with the finding that HrcQ_C_ interacts with the full-length HrcQ protein and with itself (Figure 5). It remains to be investigated whether HrcQ and HrcQ_C_ form a heterotrimeric complex as was reported for the full-length and C-terminal translation products of *spaO* (Bernal et al., 2019), *yscQ* (Bzymek et al., 2012) and *spa33* (McDowell et al., 2016). HrcQ_C_ contains a SPOA (surface presentation of antigen) domain which was also identified in SctQ proteins from animal-pathogenic bacteria and is likely involved in protein–protein interactions including homo-dimerization (Bzymek et al., 2012; Notti et al., 2015; McDowell et al., 2016; Bernal et al., 2019; Lara-Tejero et al., 2019).

To localize HrcQ_C_ and to analyze whether it is required for the assembly of HrcQ complexes, we performed fluorescence microscopy studies with HrcQ, HrcQ_M211A,_ and HrcQ_C_ as fusion partners of sfGFP or mKOk. Our studies showed that HrcQ and HrcQ_C_ colocalize and that a HrcQ_C_-sfGFP fusion did not form fluorescent foci in the absence of the full-length HrcQ protein (Figure 8). This suggests that the integration of HrcQ_C_ into the predicted sorting platform depends on HrcQ. Given that mutation of the internal translation start site reduced but did not abolish foci formation by a fluorescent HrcQ fusion, HrcQ_C_ likely promotes the assembly or association of HrcQ complexes with the T3S system but is not an essential component of the predicted sorting platform. Thus, the contribution of HrcQ_C_ to T3S differs from that of YscQ_C_ from *Yersinia* spp. which is likely an essential structural component of the sorting platform (Diepold et al., 2015). Similarly to YscQ_C_, SpaO_C_ from *Salmonella* spp. is essential for the efficient assembly of the sorting platform of the T3S system (Lara-Tejero et al., 2019). Notably, however, SpaO_C_ associates with soluble sorting platform subcomplexes but does not colocalize with the assembled final complex, suggesting that it stabilizes SpaO prior to its integration into the sorting platform (Bernal et al., 2019; Lara-Tejero et al., 2019). We predict a similar chaperone-like function for HrcQ_C_; however, in contrast to SpaO_C_, HrcQ_C_ remains associated with the predicted sorting platform and might facilitate its assembly or docking to the membrane-spanning secretion apparatus.

Previous analysis of HrcQ-sfGFP in different *X. campestris* pv. *vesicatoria* mutant strains revealed that the HrcQ complex likely associates with the IM ring component HrcD *via* the linker protein HrpB4, which acts similarly to SctK proteins from animal pathogens. HrpB4 interacts with both HrcQ and HrcD and contributes to foci formation by HrcQ-sfGFP (Otten and Büttner, 2021). The interaction of HrpB4 with HrcD depends on the cytoplasmic N-terminal domain of HrcD which presumably provides the docking site for HrpB4 (Otten and Büttner, 2021). In contrast to the essential role of SctK proteins from animal-pathogenic bacteria, however, HrpB4 only contributes to the docking of HrcQ complexes to the T3S system, suggesting that HrcQ can directly associate with HrcD in the absence of HrpB4 (Otten and Büttner, 2021). In agreement with this hypothesis, we previously detected an interaction between HrcQ and HrcD which likely depends on the cytoplasmic domain of HrcD (Otten and Büttner, 2021). In the present study, we observed that both HrcD and HrpB4 also interact with HrcQ_C_, suggesting that HrcQ_C_ contributes to the association of the predicted sorting platform with the T3S system (Figure 6).

The docking of HrcQ complexes to the T3S system depends on HrcD because HrcQ-sfGFP accumulates in the cytoplasm resulting in one bright fluorescent spot in the absence of HrcD or the cytoplasmic domain of HrcD (Otten and Büttner, 2021; Figure 8). Given that HrcQ and HrcQ_C_ still colocalize in *hrcD* mutant strains, the assembly of the predicted HrcQ-HrcQ_C_-containing complex does not depend on the association of HrcQ with the IM ring. We, therefore, assume that HrcQ_C_ is an integral component of the predicted cytoplasmic sorting platform, even when it is detached from the T3S system. Taken together, we conclude from our findings that HrcQ_C_ stabilizes HrcQ and integrates together with HrcQ into the predicted cytoplasmic sorting platform.

## Data Availability Statement

The original contributions presented in the study are included in the article/Supplementary Material, further inquiries can be directed to the corresponding author.

## Author Contributions

CO, TS, JH, and DB designed and performed the research and analyzed the data. CO wrote the manuscript and reviewed the text. DB wrote the manuscript and obtained the funding. All authors contributed to the article and approved the submitted version.

## Funding

This study was supported by grants from the Deutsche Forschungsgemeinschaft (BU2145/9-1 and BU2145/10-1) to DB.

## Conflict of Interest

The authors declare that the research was conducted in the absence of any commercial or financial relationships that could be construed as a potential conflict of interest.

## Publisher’s Note

All claims expressed in this article are solely those of the authors and do not necessarily represent those of their affiliated organizations, or those of the publisher, the editors and the reviewers. Any product that may be evaluated in this article, or claim that may be made by its manufacturer, is not guaranteed or endorsed by the publisher.
