# Supplementary material for: The Contribution of the Predicted Sorting Platform Component HrcQ to Type III Secretion in Xanthomonas campestris pv. vesicatoria Depends on an Internal Translation Start Site
Source: Front Microbiol. 2021 Oct 14;12:752733. doi: 10.3389/fmicb.2021.752733 (PMC8553256; doi:10.3389/fmicb.2021.752733)
Supplement: Supplementary file 1 [file Data_Sheet_1.pdf]

Supplemental methods

Generation of modular T3S gene cluster constructs

For the generation of the *hrcQ*$_{M211A}$*-sfgfp* module, pAGB250 (*hrcQ-sfgfp*) was amplified by PCR using two complementary primers (hrcQ-M211A-F and hrcQ-M211A-R) which introduced the M211A mutation. After *Dpn*I digest, the PCR amplicon was transferred to *E. coli*, resulting in pAGB1094. The *hrcQ*$_{M211A}$*-sfgfp* module was subsequently assembled using *Bsa*I and ligase with the inserts of pAGB249 (*PhrpD*) and pAGB231 (*Xcv term*) in the destination vector pICH47781, resulting in pAGB1095. The inserts of pAGB1095 (*phrpD-hrcQ*$_{M211A}$*-sfgfp-term*), pAGB157 (*hpaH+xopB*), pAGB160 (*hrpX*) and pAGB163 (*hrpG**) were assembled with a dummy module (pICH54022) and an end linker (pICH50881) in the *Bpi*I sites of the level M vector pAGM8079, resulting in the level M construct pAGB1096. The level M module of pAGB1096 was finally combined with the level M module of pAGB273 containing the *hrp* gene cluster with a deletion in *hrcQ* and an end-linker module (pICH79264) in the level P vector pICH75322 using *Bsa*I and ligase, resulting in pAGB1097.

For the generation of a module encoding HrcQ$_C$-sfGFP, *hrcQ*$_C$ was amplified by PCR using primers MoClo-hrcQc-N-term-F and MoClo-hrcQc-N-term-R and subcloned into the *Bpi*I sites of level -2 vector pAGM9121, generating pAGB1147. The insert of pAGB1147 was subsequently transferred to the level -1 vector pAGM1311 using *Bsa*I and ligase. This led to pAGB1148, which allowed the subsequent fusion of *hrcQ*$_C$ with genes encoding C-terminal fusion partners. For the generation of the sfGFP-encoding module, *sfgfp* was amplified with primers MoClo-CsfGFP-woLinker-F and MoClo-CsfGFP-woLinker-R and subcloned as blunt-end fragment into pICH41021, resulting in pAGB997 (CTM *sfgfp*). *hrcQ*$_C$ (pAGB1148), a linker sequence (2 × AKLEGPAGL) encoded by pAGB1000 and *sfgfp* (pAGB 997) were subsequently cloned using *Bpi*I and ligase into the level 0 vector pICH41308, resulting in pAGB1166 which contains *hrcQ*$_C$*-2×Linker-sfgfp*. pAGB1000 was generated by inserting the annealed oligonucleotides MoClo-LinkerDinh-5-3 and MoClo-LinkerDinh-3-5, which contain the linker-encoding sequence and flanking *Bpi*I sites into pICH41021, using *Sma*I and ligase. The inserts of pAGB1166 (*hrcQ*$_C$*-2×Linker-sfgfp*), pAGB249 (*PhrpD*) and pAGB231 (*Xcv term*) were ligated into the level 1 destination vector pICH47781, using *Bsa*I and ligase and resulting in the level 1 construct pAGB1167 (*PhrpD hrcQ*$_C$*-2×Linker-sfgfp*). The corresponding insert was ligated with the inserts of pAGB157 (*hpaH+xopB*), pAGB160 (*hrpX*), pAGB163 (*hrpG**), pICH54022 (dummy pos.2') and pICH50881 (end linker) into the *Bpi*I sites of the level M vector pAGM8079, resulting in the level M construct pAGB1178. For the generation of the final level P construct, the insert of pAGB1178 was ligated with the level M module of pAGB273 (contains the *hrp* gene cluster with a deletion in *hrcQ*) and an end-linker (pICH79264) into the level P vector pICH75322, using *Bsa*I and ligase and leading to pAGB1195.

For the analysis of a HrcQ$_C$-mKOκ fusion, *mKOκ* was amplified with primers MoClo-CTM-mKOκ-F and MoClo-CTM-mKOκ-R and subcloned using *Sma*I and ligase into pICH41021, thus generating pAGB1153 (CTM *mKOκ*). As described above for the generation of modules encoding HrcQ$_C$-sfGFP, the inserts of pAGB1148 (NTM *hrcQ*$_C$), pAGB1000 (2 × AKLEGPAGL) and pAGB1153 (CTM *mKOκ*) were ligated into the *Bpi*I sites of pICH41308, leading to the level 0 construct pAGB1190 (*hrcQ*$_C$*-2×Linker- mKOκ*). The insert of pAGB1190 was subsequently ligated with the inserts of pAGB249 (*PhrpD*) and pAGB231 (*Xcv term*) into the *Bsa*I sites of the level 1 destination vector pICH47742, resulting in the level 1 construct pAGB1191 (*PhrpD hrcQ*$_C$*-2×linker-mKOκ*). To generate the level M construct, the inserts of pAGB1095 (*hrcQ(M211A)-sfgfp*), pAGB1191 (*PhrpD hrcQ*$_C$*-2×linker-mKOκ*), pAGB157 (*hpaH+xopB*), pAGB160 (*hrpX*), pAGB163 (*hrpG**) and pICH50881 (end linker) were ligated into pAGM8079 using *Bpi*I and ligase and resulting in the level M construct pAGB1192. Ligation of the resulting insert with the inserts of pAGB273 (contains the *hrp* gene cluster with a deletion in *hrcQ*) and pICH79264 (end linker) into the *Bsa*I sites of pICH75322 led to the level P construct pAGB1193, which contained *hrcQ*$_C$*-2×linker-mKOκ* and *hrcQ*$_{M211A}$*-sfgfp* at positions 2' and 6 each located downstream of the native *hrcQ* promoter. All constructs are listed in Table S1 and level P constructs are summarized in Fig. S6.

To generate a module containing *hrcD*$_{\Delta 2\text{-}92}$, downstream and upstream regions were amplified by PCR using the primer pairs MoClo-DhrcD-F1/MoClo-hrcD-D2-92-R1 and MoClo-hrcD-D2-

92-F3/MoClo-DhrcD-R3 and pAGB206 as template, which contains *hpaA* and *hrcD*. Both amplicons were assembled using *Bpi*I and ligase in the level -2 vector pAGM9121, resulting in pAGB1206 (*hpaA*, $hrcD_{\Delta 2\text{-}92}$). The insert of pAGB1206 was subsequently assembled with the inserts of pAGB205 (*hrcS*), pAGB207 (*hrpD6*), pAGB208 (*hrpE*-*hpaB*) and pAGB209 (*hpaE*) in the level -1 vector pAGM1311 using *Bsa*I and ligase and resulting in pAGB1207 (*hrcS*-*hpaA*, $hrcD_{\Delta 2\text{-}92}$-*hrpD6*-*hrpE*, *hpaB*-*hpaE*). The insert of pAGB1207 was subsequently assembled with the insert of pAGB272 (*hrpC* operon, Δ*hrcQ*, *hrcR*) in the *Bpi*I sites of level 0 vector pICH41331, thus generating the level 0 construct pAGB1209 which contains the *hrpC*, *hrpE* and *hpaB* operons as well as the *hrpD* operon with a deletion in *hrcQ* and in codons 2 - 92 of *hrcD*. The insert of construct pAGB1209 was subsequently transferred into the level 1 destination vector pICH47751 using *Bsa*I and ligase, thus generating the level 1 construct pAGB1211. The insert of pAGB1211 was assembled with the inserts of pAGB154 (*hrpA* and *hrpB* operons), pAGB156 (*hrpF*), the dummy module pICH54011 and the end linker pICH50900 into pAGM8031 using *Bpi*I and ligase, leading to the level M construct pAGB1216 (*hrp* gene cluster with a deletion in *hrcQ* and containing $hrcD_{\Delta 2\text{-}92}$). The final level P construct was generated by assembly of the inserts of pAGB322 (*hrcQ-sfgfp*), pAGB1216 (*hrp* gene cluster with a deletion in *hrcQ* and containing $hrcD_{\Delta 2\text{-}92}$) and the end linker pICH79264 in pICH75322, using *Bsa*I and ligase and resulting in the level P construct pAGB1217. For colocalization studies with $HrcQ_{M211A}$-sfGFP and $HrcQ_C$-2×Linker-mKOκ, the level M constructs pAGB1216 (*hrp* gene cluster with a deletion in *hrcQ* and containing $hrcD_{\Delta 2\text{-}92}$) and pAGB1192 ($hrcQ_{M211A}$-*sfgfp* + $hrcQ_C$-2×*Linker-mKOκ*) were assembled with an end linker (construct pICH79264) in pICH75322 using *Bsa*I and ligase, thus generating the level P construct pAGB1226.

Table S1: Bacterial strains and plasmids used in this study.

Strain or plasmid	Relevant characteristics	Reference(s)
**Strains**		
X. campestris pv. vesicatoria		
85-10	Pepper-race 2; wild type; Rif^R	Canteros, 1990; Kousik and Ritchie, 1998
85*	85-10 derivative containing the *hrpG** mutation	Wengelnik et al., 1999
85-10Δ*hrcQ*	85-10 derivative deleted in codons 11 - 243 of *hrcQ* followed by a nonsense mutation	Lorenz et al., 2012
85*Δ*hrcQ*	85* derivative deleted in codons 11 - 243 of *hrcQ* followed by a nonsense mutation	Lorenz et al., 2012
85-10Δ*hrcQ*::*hrcQ*	Derivative of strain 85-10Δ*hrcQ* carrying *hrcQ-c-myc* under control of the native promoter inserted into the *hpaFG* region	Lorenz et al., 2012
85*Δ*hrcQ*::*hrcQ*	Derivative of strain 85*Δ*hrcQ* carrying *hrcQ-c-myc* under control of the native promoter inserted into the *hpaFG* region	Lorenz et al., 2012
85-10Δ*hrcQ*::$hrcQ_{M211A}$	Derivative of strain 85-10Δ*hrcQ* carrying $hrcQ_{M211A}$-*c-myc* under control of the native promoter inserted into the *hpaFG* region	This study
85*Δ*hrcQ*::$hrcQ_{M211A}$	Derivative of strain 85*Δ*hrcQ* carrying $hrcQ_{M211A}$-*c-myc* under control of the native promoter inserted into the *hpaFG* region	This study
85*Δ*hrp*_fs*HAGX*	Derivative of strain 85*Δ*hrp* with frameshift mutations after codons 26 of *hrpX*, 12 of *hrpG*, 16 of *xopA* and 7 of *hpaH*	Hausner et al., 2019
E.coli		
OneShot®TOP10	F^-,*mcrA*Δ*(mrr-hsdRMS-mcrBC)*, Φ*80lacZ*Δ*M15*, Δ*lacX74*, *recA1*, *ara*Δ*139*Δ*(ara-leu)7697*, *galU*, *galK*, *rpsL*, *endA1*, *nupG*	Invitrogen
BL21 (DE3)	F^-, *ompT. hsdSB* (rB^- mB^-), *gal*, *dcm* (DE3)	Stratagene
JM109	F^-, *traD36* $proA^+B^+$ $lacI^q$ *Δ(lacZ)M15/ Δ(lac-proAB) glnV44* $e14^-$ *gyrA96 recA1 relA1 endA1 thi hsdR17*	Yanisch-Perron et al., 1985
DHM1	F^-, *cya-854*, *recA1*, *endA1*, *gyrA96* (Nal^R), *thi1*, *hsdR17*, *spoT1*, *rfbD1*, *glnV44(AS)*	Karimova et al., 2005
BTH101	F^-, *cya-99*, *araD139*, *galE15*, *galK16*, *rpsL1* (Str^R), *hsdR2*, *mcrA1*, *mcrB1*	Euromedex; Battesti and Bouveret, 2012
**Plasmids**		
pRK2013	ColE1 replicon, $TraRK^+$ Mob^+; Km^R	Figurski and Helinski, 1979
pICH41021	Derivative of pUC19 with mutated *Bsa*I site; Ap^R	Gift from S. Marillonnet
pBRM	Golden Gate-compatible derivative of pBBR1MCS-5 containing the *lac* promoter, a *lacZα* fragment flanked by *Bsa*I recognition sites and a 3 x c-Myc epitope-encoding sequence; Gm^R	Szczesny et al., 2010
pBRM-P	Derivative of pBRM lacking the *lac* promoter upstream of the 5' *Bsa*I site	Szczesny et al., 2010
pBRM-P-stop	Derivative of pBRM-P with a stop codon upstream of the 3 x c-Myc epitope-encoding sequence	Hausner et al., 2017

pLAND-P	Derivative of pOK1 carrying fragments of the *hpaFG* region flanking a *lacZα* fragment, the *lac* promoter and a 3 x c-Myc epitope-encoding sequence	Lorenz et al., 2012
pBhrcQ	Derivative of pBRM encoding HrcQ-c-Myc	Lorenz et al., 2012
pBhrcQstop	Derivative of pBRM encoding HrcQ	Lorenz et al., 2012
pB-PhrcQ	Derivative of pBRM-P containing *hrcQ-c-myc* and 299 bp upstream region	Lorenz et al., 2012
pB-PhrcQstop	Derivative of pBRM-P containing *hrcQ* and 299 bp upstream region	Lorenz et al., 2012
pB-PhrcQstop$_{V1A}$	Derivative of pBRM-P containing *hrcQ*$_{V1A}$ (mutation of the GTG codon to GCG) and 299 bp upstream region	This study
pB-PhrcQstop$_{L+13A}$	Derivative of pBRM-P containing *hrcQ*$_{L+13A}$ (mutation of the TTG codon to GCG) and 299 bp upstream region	This study
pB-PhrcQstop$_{L+30A}$	Derivative of pBRM-P containing *hrcQ*$_{L+30A}$ (mutation of the TTG codon to GCG) and 299 bp upstream region	This study
pB-PhrcQstop$_{V1A/L+13A}$	Derivative of pBRM-P containing *hrcQ*$_{V1A/L+13A}$ and 299 bp upstream region	This study
pB-PhrcQstop$_{V1A/L+30A}$	Derivative of pBRM-P containing *hrcQ*$_{V1A/L+30A}$ and 299 bp upstream region	This study
pB-PhrcQstop$_{L+13A/L+30A}$	Derivative of pBRM-P containing *hrcQ*$_{L+13A/L+30A}$ and 299 bp upstream region	This study
pB-PhrcQstop$_{V1A/L+13A/L+30A}$	Derivative of pBRM-P containing *hrcQ*$_{V1A/L+13A/L+30A}$ and 299 bp upstream region	This study
pBhrcQ$_{M211A}$	Derivative of pBRM encoding HrcQ$_{M211A}$-c-Myc	This study
pB-PhrcQ$_{M203A}$	Derivative of pBRM-P containing *hrcQ*$_{M203A}$*-c-myc* and 299 bp upstream region	This study
pB-PhrcQ$_{M211A}$	Derivative of pBRM-P containing *hrcQ*$_{M211A}$*-c-myc* and 299 bp upstream region	This study
pLAND-PhrcQ$_{M211A}$	Derivative of pLAND-P containing *hrcQ*$_{M211A}$*-c-myc* and 299 bp upstream region	This study
pB-PhrcQ-sfGFPstop	Derivative of pBRM-P containing *hrcQ-sfgfp* and 299 bp upstream region	This study
pB-PhrcQ$_{M203A}$-sfGFPstop	Derivative of pBRM-P containing *hrcQ*$_{M203A}$*-sfgfp* and 299 bp upstream region	This study
pB-PhrcQ$_{M211A}$-sfGFPstop	Derivative of pBRM-P containing *hrcQ*$_{M211A}$*-sfgfp* and 299 bp upstream region	This study
pBhrcQ$_C$	Derivative of pBRM encoding HrcQ$_C$-c-Myc	This study
pB-PhrcQ$_C$	Derivative of pBRM-P containing *hrcQ*$_C$*-c-myc* and 299 bp upstream region of *hrcQ*	This study
pB-P-stop-ptacGST-hrcQ$_C$	Derivative of pBRM-P-stop encoding GST-HrcQ$_C$ under control of the *ptac* promoter	This study
pEX-A sfgfp	Derivative of pEX-A containing *sfgfp* with two stop codons and an N-terminal linker (AKLEGPAGL) flanked by GCTA/GGTG *Bsa*I fusion sites	Synthezised by Eurofins Genomics GmbH
**BACTH vectors**		
pUT18	BACTH vector, derivative of pUC19, encodes the T18 fragment (amino acids 225 – 399) of CyaA downstream of a *lac* promoter and a multiple cloning site for classical cloning, Ap^r	Euromedex; Karimova et al., 2001

pUT18C	BACTH vector, derivative of pUT18, encodes the T18 fragment (amino acids 225 – 399) of CyaA downstream of a *lac* promoter; the multiple cloning site for classical cloning is inserted at the 3' end of the T18-encoding fragment, Ap^r	Euromedex; Karimova et al., 2001
pUT18$_{GG}$	Golden Gate-compatible derivative of pUT18 containing *lacP-eforRed* flanked by *Bsa*I sites upstream of the *FLAG-T18* fragment; Gm^R	Otten and Büttner, 2021
pUT18C$_{GG}$	Golden Gate-compatible derivative of pUT18C containing *lacP-eforRed* flanked by *Bsa*I sites downstream of the *T18-FLAG* fragment; Gm^R	Otten and Büttner, 2021
pKT25	BACTH vector, derivative of low copy number plasmid pSU40, encodes the T25 fragment (first 224 amino acids) of CyaA downstream of a *lac* promoter, multiple cloning site for classical cloning is inserted at the 3' end of the T25 encoding fragment, Km^r	Euromedex; Karimova et al., 2001
pKNT25	BACTH vector, derivative of pKT25, contains the T25-encoding fragment downstream of a multiple cloning site for classical cloning and the *lac* promoter, Km^r	Euromedex; Karimova et al., 2001
pKT25$_{GG}$	Golden Gate-compatible derivative of pKT25 encoding the T25 fragment downstream of a *lac* promoter and in frame with a C-terminal FLAG epitope-encoding sequence; contains *lacP-eforRed* flanked by *Bsa*I sites downstream of the *T25-FLAG* fragment; Km^r	Otten and Büttner, 2021
pKTN25$_{GG}$	Golden Gate-compatible derivative of pKNT25, encodes the T25 fragment in frame with an N-terminal FLAG epitope-encoding sequence downstream of the *lac* promoter, contains *lacP-eforRed* flanked by *Bsa*I sites upstream of the *T25-FLAG* fragment; Km^r	Otten and Büttner, 2021
pUT18$_{GG}$-hrcQ	Derivative of pUT18$_{GG}$ encoding HrcQ-FLAG-T18	Otten and Büttner, 2021
pUT18C$_{GG}$-hrcQ	Derivative of pUT18C$_{GG}$ encoding T18-FLAG-HrcQ	Otten and Büttner, 2021
pKT25$_{GG}$-hrcQ	Derivative of pKT25$_{GG}$ encoding T25-FLAG-HrcQ	Otten and Büttner, 2021
pKNT25$_{GG}$-hrcQ	Derivative of pKTN25$_{GG}$ encoding HrcQ-FLAG-T25	Otten and Büttner, 2021
pUT18$_{GG}$-hrcQ$_{M211A}$	Derivative of pUT18$_{GG}$ encoding HrcQ$_{M211A}$-FLAG-T18	This study
pUT18C$_{GG}$-hrcQ$_{M211A}$	Derivative of pUT18C$_{GG}$ encoding T18-FLAG-HrcQ$_{M211A}$	This study
pKT25$_{GG}$-hrcQ$_{M211A}$	Derivative of pKT25$_{GG}$ encoding T25-FLAG-HrcQ$_{M211A}$	This study
pKNT25$_{GG}$-hrcQ$_{M211A}$	Derivative of pKT25$_{GG}$ encoding HrcQ$_{M211A}$-FLAG-T25	This study
pUT18$_{GG}$-hrcQ$_{C}$	Derivative of pUT18$_{GG}$ encoding HrcQ$_{C}$-FLAG-T18	This study
pUT18C$_{GG}$-hrcQ$_{C}$	Derivative of pUT18C$_{GG}$ encoding T18-FLAG-HrcQ$_{C}$	This study
pKT25$_{GG}$-hrcQ$_{C}$	Derivative of pKT25$_{GG}$ encoding T25-FLAG-HrcQ$_{C}$	This study
pKNT25$_{GG}$-hrcQ$_{C}$	Derivative of pKTN25$_{GG}$ encoding HrcQ$_{C}$-FLAG-T25	This study
pUT18$_{GG}$-hrpB4	Derivative of pUT18$_{GG}$ encoding HrpB4-FLAG-T18	Otten and Büttner, 2021

pUT18C$_{GG}$-hrpB4	Derivative of pUT18C$_{GG}$ encoding T18-FLAG-HrpB4	Otten and Büttner, 2021
pKT25$_{GG}$-hrpB4	Derivative of pKT25$_{GG}$ encoding T25-FLAG-HrpB4	Otten and Büttner, 2021
pKTN25$_{GG}$-hrpB4	Derivative of pKNT25-GG encoding HrpB4-FLAG-T25	Otten and Büttner, 2021
pUT18C$_{GG}$-hrcD	Derivative of pUT18C$_{GG}$ encoding T18-FLAG-HrcD	Otten and Büttner, 2021
pKT25$_{GG}$-hrcD	Derivative of pKT25$_{GG}$ encoding T25-FLAG-HrcD	Otten and Büttner, 2021
**Constructs for modular cloning**		
Destination vectors		
pAGM9121	pUC19-derived vector, *lacZα* fragment flanked by *Bpi*I sites; Sm^R	Addgene #51833; Weber et al., 2011
pAGM1311	pUC19-derived level -1 vector, *lacZα* fragment flanked by *Bsa*I sites; Km^R	Addgene #51833; Weber et al., 2011
pICH41276	pUC19-derived level 0 vector, *lacZα* fragment flanked by *Bpi*I sites; Sm^R	Addgene #51833; Weber et al., 2011
pICH41295	pUC19-derived level 0 vector, *lacZα* fragment flanked by *Bpi*I sites; Sm^R	Addgene #51833; Weber et al., 2011
pICH41308	pUC19-derived level 0 vector, *lacZα* fragment flanked by *Bpi*I sites; Sm^R	Addgene #47998; Weber et al., 2011
pICH41331	pUC19-derived level 0 vector, *lacZα* fragment flanked by *Bpi*I sites; Sm^R	Addgene #47999; Weber et al., 2011
pICH47751	Level 1 destination vector derived from pBIN19 and pUC19, *lacZα* fragment flanked by *Bsa*I sites, for level M position 3; Ap^R	Addgene #48002; Weber et al., 2011
pICH47781	Level 1 destination vector derived from pBIN19 and pUC19, *lacZα* fragment flanked by *Bsa*I sites, for level M position 6; Ap^R	Addgene #48005; Weber et al., 2011
pAGM8031	Level M vector derived from pBIN19 and pUC19, *lacZα* fragment flanked by *Bpi*I sites; Sm^R	Addgene #48037; Weber et al., 2011
pAGM8079	Level M vector derived from pBIN19 and pUC19, *lacZα* fragment flanked by *Bpi*I sites; Sm^R	Addgene #48041; Weber et al., 2011
pICH75322	Level P vector derived from pPZP200 and pUC19, *lacZα* fragment flanked by *Bsa*I sites, ColE1 and pVS1 ori, Km^R	Addgene #48051; Weber et al., 2011
End-linker constructs		
pICH79264	Derived from pUC19, level P end linker for position 3; ACTA / GGGA *Bsa*I and ACTA/- *Bpi*I fusion sites; Ap^R	Addgene #48059; Weber et al., 2011
pICH50881	Derived from pUC19, level M end linker for position 3; ACTA/- *Bsa*I and ACTA/ GGGA *Bpi*I fusion sites; Ap^R,	Addgene #48045; Weber et al., 2011
pICH50900	Derived from pUC19, level M end linker for position 5; CAGA/ - *Bsa*I and CAGA/ GGGA *Bpi*I fusion sites; Ap^R	Addgene #48047; Weber et al., 2011
Dummy modules		
pICH54011	Derived from pBIN19 and pUC19, 15-bp insert for level M position 1 with TGCC/ GCAA *Bpi*I fusion sites; Ap^R	Addgene #48065; Weber et al., 2011
pICH54022	Derived from pBIN19 and pUC19, 15-bp insert for level M position 2' with GCAA/ ACTA *Bpi*I fusion sites; Ap^R	Addgene #48066; Weber et al., 2011
pICH54066	Derived from pBIN19 and pUC19, 15-bp insert for level M position 6 with TGTG/ GAGC *Bpi*I fusion sites; Ap^R	Addgene #48070; Weber et al., 2011

Level -2 constructs		
pAGB205	Level -2 construct; derivative of pAGM9121 containing *hrcS*; Sm^R	Hausner et al., 2019
pAGB206	Level -2 construct; derivative of pAGM9121 containing *hpaA* and *hrcD*; Sm^R	Hausner et al., 2019
pAGB207	Level -2 construct; derivative of pAGM9121 containing *hrpD6*; Sm^R	Hausner et al., 2019
pAGB208	Level -2 construct; derivative of pAGM9121 containing *hrpE* and *hpaB*; Sm^R	Hausner et al., 2019
pAGB209	Level -2 construct; derivative of pAGM9121 containing *hpaE*; Sm^R	Hausner et al., 2019
pAGB1147	Level -2 construct; derivative of pAGM9121 containing *hrcQ*$_C$ without stop codon; Sm^R	This study
pAGB1206	Level -2 construct; derivative of pAGM9121 containing *hpaA* and *hrcD*$_{\Delta 2\text{-}92}$ (deletion of bp 4 - 276 of *hrcD*); Sm^R	This study
Level -1 constructs		
pAGB272	Level -1 construct; derivative of pAGM1311 containing *hrcU*, *hrcV*, *hpaC*, *hrpD6*, Δ*hrcQ*, *hrcR*; Km^R	Hausner et al., 2019
pAGB997	Level -1 construct; derivative of pICH41021 containing *sfgfp* for generation of C-terminal fusions; Ap^R	This study
pAGB1000	Level -1 construct; derivative of pICH41021 containing a linker (2 x AKLEGPAGL)-encoding sequence; Ap^R	This study
pAGB1148	Level -1 construct; derivative of pAGM1311 containing *hrcQ*$_C$ without stop codon for generation of C-terminal fusions; Km^R	This study
pAGB1153	Level -1 construct; derivative of pICH41021 containing *mKO*$_\kappa$ for generation of C-terminal fusions; Ap^R	This study
pAGB1207	Level -1 construct; derivative of pAGM1311 containing *hrcS*, *hpaA*, *hrcD*$_{\Delta 2\text{-}92}$ mutant, *hrpD6*, *hrpE*, *hpaB*, *hpaE*; Km^R	This study
Level 0 constructs		
pAGB231	Level 0 construct; derivative of pICH41276 containing a transcriptional terminator; Sm^R	Hausner et al., 2019
pAGB249	Level 0 construct; derivative of pICH41295 containing the native *hrpD* operon promoter; Sm^R	Hausner et al., 2019
pAGB250	Level 0 construct; derivative of pICH41308 encoding HrcQ-sfGFP with a linker (AKLEGPAGL); Sm^R	Hausner et al., 2019
pAGB1094	Level 0 construct; derivative of pICH41308 encoding HrcQ$_{M211A}$-sfGFP with a linker (AKLEGPAGL); Sm^R	This study
pAGB1166	Level 0 construct; derivative of pICH41308 containing *hrcQ*$_C$*-2xAKLEGPAGL-sfgfp*; Sm^R	This study
pAGB1190	Level 0 construct; derivative of pICH41308 encoding HrcQ$_{M211A}$-mKO$_\kappa$; Sm^R	This study
pAGB1209	Level 0 construct; derivative of pICH41331 containing the *hrpC*, *hrpD*, *hrpE* and *hpaB* operons operons with a deletion in *hrcQ* and codons 2 - 92 of *hrcD*; Sm^R	This study

Level 1 constructs		
pAGB154	Level 1 construct; derivative of pICH47811 containing the *hrpA* and *hrpB* operons; Ap^R	Hausner et al., 2019
pAGB156	Level 1 construct; derivative of pICH47761 containing the *hrpF* operon; Ap^R	Hausner et al., 2019
pAGB157	Level 1 construct; derivative of pICH47772 containing *xopA* and *hpaH*; Ap^R	Hausner et al., 2019
pAGB160	Level 1 construct; derivative of pICH47861 containing *hrpX*; Ap^R	Hausner et al., 2019
pAGB163	Level 1 construct; derivative of pICH47732 containing *hrpG**; Ap^R	Hausner et al., 2019
pAGB1095	Level 1 construct; derivative of pICH47781 encoding $HrcQ_{M211A}$-sfGFP under control of the native *hrpD* operon promoter; Ap^R	This study
pAGB1167	Level 1 construct; derivative of pICH47781 containing $hrcQ_C$*-2xAKLEGPAGL-sfgfp* downstream of the native *hrpD* operon promoter; Ap^R	This study
pAGB1191	Level 1 construct; derivative of pICH47781 containing $hrcQ_C$*-2xAKLEGPAGL-*mKO_κ downstream of the native *hrpD* operon promoter; Ap^R	This study
pAGB1211	Level 1 construct; derivative of pICH47751 containing the *hrpC*, *hrpD*, *hrpE* and *hpaB* operons with a deletion in *hrcQ* and $hrcD_{\Delta 2\text{-}92}$; Ap^R	This study
Level M constructs		
pAGB273	Level M construct; derivative of pAGM8031 containing the *hrp* gene cluster deleted in *hrcQ*; Sm^R	Hausner et al., 2019
pAGB278	Level M construct; derivative of pAGM8079 containing *xopA*, *hpaH*, *hrpX* and *hrpG**; Sm^R	This study
pAGB322	Level M construct; derivative of pAGM8079 containing *xopA*, *hpaH*, *hrcQ-sfgfp*, *hrpX* and *hrpG**; Sm^R	Hausner et al., 2019
pAGB1096	Level M construct; derivative of pAGM8079 containing *xopA*, *hpaH*, $hrcQ_{M211A}$*-sfgfp*, *hrpX* and *hrpG**; Sm^R	This study
pAGB1178	Level M construct; derivative of pAGM8079 containing *xopA*, *hpaH*, $hrcQ_C$*-2xAKLEGPAGL-sfgfp*, *hrpX* and *hrpG**; Sm^R	This study
pAGB1192	Level M construct; derivative of pAGM8079 containing *xopA*, *hpaH*, $hrcQ_{M211A}$*-sfgfp*, *hrpX*, *hrpG** and $hrcQ_C$*-2xAKLEGPAGL-*mKO_κ; Sm^R	This study
pAGB1216	Level M construct; derivative of pAGM8031 containing the *hrp* gene cluster with $hrcD_{\Delta 2\text{-}92}$ and a deletion in *hrcQ*; Sm^R	This study
Level P constructs		
pAGB324	Level P construct; derivative of pICH75322 containing the *hrp* gene cluster (with a deletion in *hrcQ*), *xopA*, *hpaH*, *hrcQ-sfgfp*, *hrpX* and *hrpG**; Km^R	Hausner et al., 2019
pAGB866	Level P construct; derivative of pICH75322 containing the *hrp* gene cluster (with deletions in *hrcQ* and *hrcD*), *xopA*, *hpaH*, *hrcQ-sfgfp*, *hrpX* and *hrpG**; Km^R	Otten and Büttner, 2021

pAGB1097	Level P construct; derivative of pICH75322 containing the *hrp* gene cluster (with deletion in *hrcQ*), *xopA*, *hpaH*, *hrcQ*$_{M211A}$-*sfgfp*, *hrpX* and *hrpG**; Km^R	This study
pAGB1193	Level P construct; derivative of pICH75322 containing the *hrp* gene cluster (with a deletion in *hrcQ*), *xopA*, *hpaH*, *hrcQ*$_{M211A}$-*sfgfp*, *hrpX*, *hrpG** and *hrcQ*$_C$-*2xAKLEGPAGL-mKO*$_\kappa$; Km^R	This study
pAGB1195	Level P construct; derivative of pICH75322 containing the *hrp* gene cluster (with a deletion in *hrcQ*), *xopA*, *hpaH*, *hrcQ*$_C$-*2xAKLEGPAGL-sfgfp*, *hrpX* and *hrpG**; Km^R	This study
pAGB1217	Level P construct; derivative of pICH75322 containing the *hrp* gene cluster (with *hrcD*$_{\Delta 2\text{-}92}$ and a deletion of *hrcQ*), *xopA*, *hpaH*, *hrcQ-sfgfp*, *hrpX*, *hrpG**; Km^R	This study
pAGB1226	Level P construct; derivative of pICH75322 containing the *hrp* gene cluster (with *hrcD*$_{\Delta 2\text{-}92}$ and a deletion of *hrcQ*), *xopA*, *hpaH*, *hrcQ*$_{M211A}$-*sfgfp*, *hrpX*, *hrpG** and *hrcQ*$_C$-*2xAKLEGPAGL-mKO*$_\kappa$; Km^R	This study

References

Battesti, A., and Bouveret, E. (2012). The bacterial two-hybrid system based on adenylate cyclase reconstitution in *Escherichia coli*. Methods *58*, 325-334.

Canteros, B.I. (1990). Diversity of plasmids and plasmid-encoded phenotypic traits in *Xanthomonas campestris* pv. *vesicatoria*. PhD thesis, University of Florida.

Figurski, D., and Helinski, D.R. (1979). Replication of an origin-containing derivative of plasmid RK2 dependent on a plasmid function provided *in trans*. Proc Nat Acad Sci USA *76*, 1648-1652.

Hausner, J., Hartmann, N., Jordan, M., and Büttner, D. (2017). The predicted lytic transglycosylase HpaH from *Xanthomonas campestris* pv. *vesicatoria* associates with the type III secretion system and promotes effector protein translocation. Infect Immunol *85, e00788-16*.

Hausner, J., Jordan, M., Otten, C., Marillonnet, S., and Buttner, D. (2019). Modular cloning of the type III secretion gene cluster from the plant-pathogenic bacterium *Xanthomonas euvesicatoria*. ACS Synth Biol *8*, 532-547.

Karimova, G., Dautin, N., and Ladant, D. (2005). Interaction network among *Escherichia coli* membrane proteins involved in cell division as revealed by bacterial two-hybrid analysis. J Bacteriol *187*, 2233-2243.

Karimova, G., Ullmann, A., and Ladant, D. (2001). Protein-protein interaction between *Bacillus stearothermophilus* tyrosyl-tRNA synthetase subdomains revealed by a bacterial two-hybrid system. J Mol Microbiol Biotechnol *3*, 73-82.

Kousik, C.S., and Ritchie, D.F. (1998). Response of bell pepper cultivars to bacterial spot pathogen races that individually overcome major resistance genes. Plant Disease *82*, 181-186.

Lorenz, C., Hausner, J., and Büttner, D. (2012). HrcQ provides a docking site for early and late type III secretion substrates from *Xanthomonas*. PLoS ONE *7*, e51063.

Otten, C., and Büttner, D. (2021). HrpB4 from *Xanthomonas campestris* pv. *vesicatoria* acts similarly to SctK proteins and promotes the docking of the predicted sorting platform to the type III secretion system. Cell Microbiol, e13327.

Szczesny, R., Jordan, M., Schramm, C., Schulz, S., Cogez, V., Bonas, U., and Büttner, D. (2010). Functional characterization of the Xps and Xcs type II secretion systems from the plant pathogenic bacterium *Xanthomonas campestris* pv. *vesicatoria*. New Phytol *187*, 983-1002.

Weber, E., Engler, C., Gruetzner, R., Werner, S., and Marillonnet, S. (2011). A modular cloning system for standardized assembly of multigene constructs. PLoS ONE *6*, e16765.

Wengelnik, K., Rossier, O., and Bonas, U. (1999). Mutations in the regulatory gene *hrpG* of *Xanthomonas campestris* pv. vesicatoria result in constitutive expression of all *hrp* genes. J Bacteriol *181*, 6828-6831.

Yanisch-Perron, C., Vieira, J., and Messing, J. (1985). Improved M13 phage cloning vectors and host strains: nucleotide sequences of the M13mp18 and pUC19 vectors. Gene *33*, 103-119.

Table S2: Primers used in this study.

Name[1]	Sequence[2]
hrcQ-BsaI_F	TTT **GGTCTC** T TATG GTGTTCGGCGACCCACGCGC
hrcQ-BsaI-R	TTT **GGTCTC** T CACC GGCATCTGCATGCGTGCTCTC
hrcQ-FP-R	TTT **GGTCTC** T TAGC GGCATCTGCATGCGTGCTCTCCG
hrcQProm300-F	TTT **GGTCTC** T ATTC CGCACAGACGACGTTGT
hrcQProm300-R	TTT **GGTCTC** T CATA CTGCCAAGCTGGGCGCGCTC
hrcQ-V1A-F	CAGCTTGGCAGAGCGTTCGGCGAC
hrcQ-V1A-R	GTCGCCGAACGCTCTGCCAAGCTG
hrcQ-L+13A-F	CTCAGGCGGCGACGCGCGTTG
hrcQ-L+13A-R	CAACGCGCGTCGCCGCCTGAG
hrcQ-L+30A-F	GAACTCCATCGCGCTAACCGAGCAG
hrcQ-L+30A-R	CTGCTCGGTTAGCGCGATGGAGTTC
hrcQ-M203A-Bpi-F	TTT **GAAGAC** TT TGGA GGCCAGCCCCACCATG
hrcQ-M203A-Bpi-R	TTT **GAAGAC** TT TCCA GGATCGCCTGTTGCAGGTTCAAGCGCACTG
hrcQ-M211A-F	CAGCCCCACCGCGCAGCACGATAC
hrcQ-M211A-R	GTATCGTGCTGCGCGGTGGGGCTG
hrcQc-F	TTT **GGTCTC** T TATG CAGCACGATACGTTTGAGCCGGAAG
MoClo-DhrcD-F1	TTT **GAAGAC** AA CTCA CCAT GATCCGTCGCATCTC
MoClo-hrcD-D2-92-R1	TTT **GAAGAC** AA CATG GGCGAACCTCCTGAGC
MoClo-hrcD-D2-92-F3	TTT **GAAGAC** AA CATG GCGCTGCTGGAGCGGCTGCTTTC
MoClo-DhrcD-R3	TTT **GAAGAC** AA CTCG TCAT TGCGCCGCTTGCTGCGGCAG
MoClo-hrcQc-NTM-F	TTT **GAAGAC** AA CTCA ACAT AATG CAGCACGATACGTTTGAGC
MoClo-hrcQc-NTM-R	TTT **GAAGAC** AA CTCG ACAA TAGC GGCATCTGCATGCGTG
MoClo-CTM-sfGFP-woLinker-F	TTT **GAAGAC** AA CCTG CGTAAAGGCGAGGAG
MoClo-CTM-sfGFP-woLinker-R	TTT **GAAGAC** AA AAGC TCATCA TTTGTACAGTTCATC
MoClo-CTM-mKOκ-F	TTT **GAAGAC** AA CCTG GTGAGTGTGATTAAACCAGAG
MoClo-CTM-mKOκ-R	TTT **GAAGAC** AA AAGC TCA CTTGTCGTCATCGTCTTTGTAGTCGGAATGAGCTACTGCATC
MoClo-LinkerDinh-5-3	TTT **GAAGAC** AA GCTA AGCTGGAAGGCCCGGCCGGCCTGGCAAAGCTAGAAGGTCCGGCAGGACT CCTG TT **GTCTTC** AAA
MoClo-LinkerDinh-3-5	TTT **GAAGAC** AA CAGG AGTCCTGCCGGACCTTCTAGCTTTGCCAGGCCGGCCGGGCCTTCCAGCT TAGC TT **GTCTTC** AAA

[1] NTM, N-terminal module, allows fusion to a reporter gene at the 3' end; CTM, C-terminal module, allows fusion to a reporter gene at the 5' end.
[2] *Bsa*I and *Bpi*I recognition sites are shown in bold.

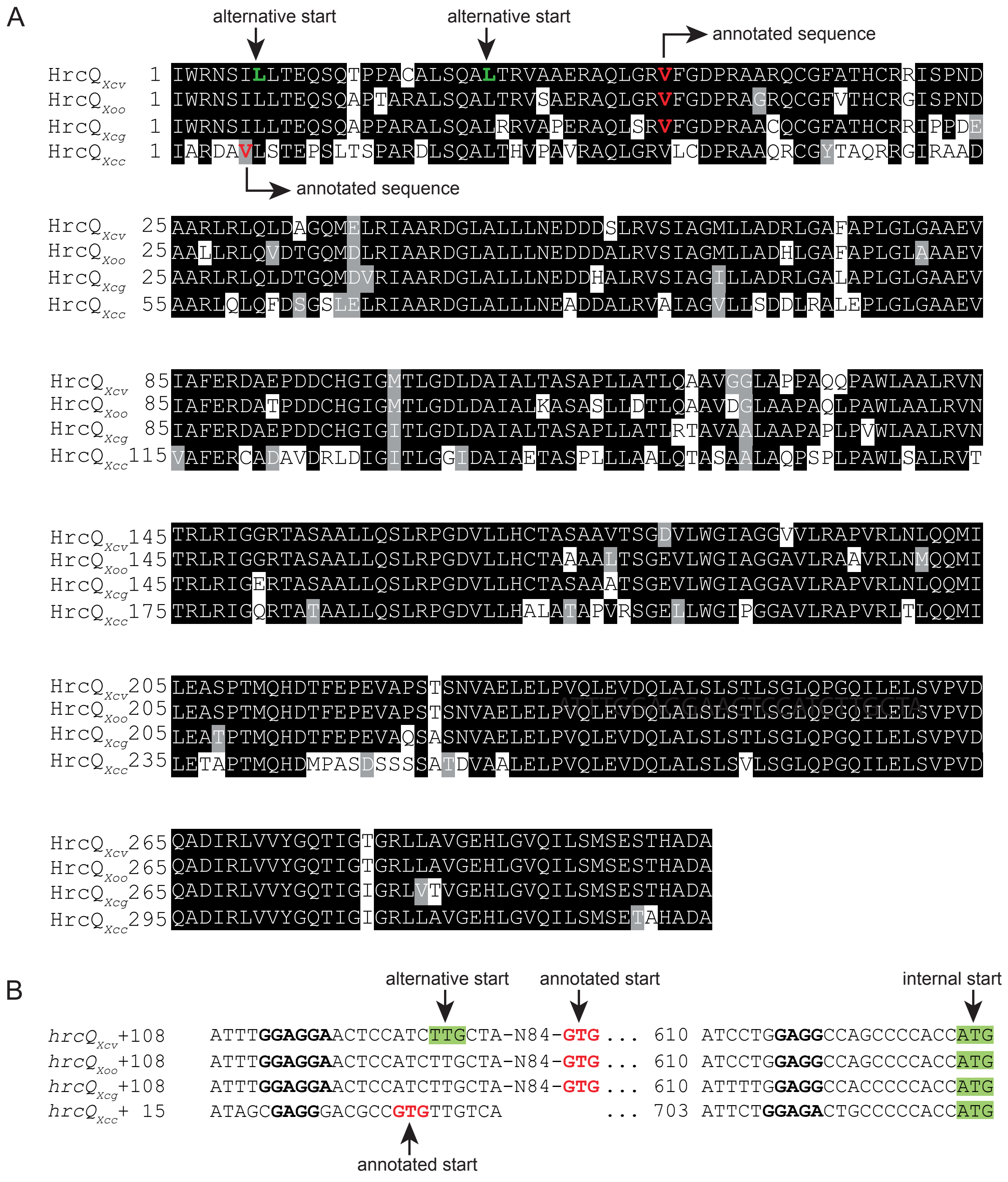

Figure S1 Sequence comparison of HrcQ proteins from *Xanthomonas* spp. and upstream DNA sequences.
(A) Alignment of HrcQ proteins from *Xanthomonas* spp. including putative amino acids encoded by sequences upstream of the annotated start sites. The first amino acids of the annotated proteins are shown in red. Amino acids encoded by putative alternative start sites in HrcQ from *X. campestris* pv. *vesicatoria* are indicated. Numbers refer to amino acid positions of the annotated proteins. The following sequences were used: HrcQ from *X. campestris* pv. *vesicatoria* (*Xcv,* GenBank accession number CAJ22054), HrcQ from *X. oryzae* pv. *oryzae (Xoo*, GenBank accession number AAK08059), HrcQ from *X. citri* pv. *glycines* (*Xcg*, GenBank accession number AAP34348) and HrcQ from *X. campestris* pv. *campestris* (*Xcc*, GenBank accession number CAP52441).
(B) Putative Shine Dalgarno sequences upstream of predicted translation start sites are conserved in *Xanthomonas* spp. *hrcQ* genes from *X. campestris* pv. *vesicatoria* (*Xcv*), *X. oryzae* pv. *oryzae (Xoo*; GenBank accession number AF320050), *X. citri* pv. *glycines* (*Xcg*, GenBank accession number AF499777) and X. *campestris* pv. *campestris* (*Xcc*; GenBank accession number AM920689) were compared. Putative Shine Dalgarno sequences are shown in bold and annotated start codons in red. Predicted start codon at codon position +30 of *hrcQ* from *X. campestris* pv. *vesicatoria* as well as at codon position 211 are indicated. Numbers refer to nucleotide positions upstream (indicated with +) and downstream of the annotated start sites.

```
HrcQPss          -MNAH------ALPLRRLSHAQVRIARRLAGEPWMDFSVSDQPGRMTLRTSRRAPASTPM
HrcQEa         1 MSEAT------PLNLPRLTRAGVRSQNRLAGAHHYPFTLGDESGALYILPGTPQ-QSVEL
HrcQXcv        1 TEQSQTPPACALSQALTRVAAERAQLGRVFGDPRAA----------------------RQ
HrcQRsol       1 -MNAGPSFSALEPHLRAFTPAHAALTRLLDGVHRGG----------------------DS

HrcQPss           FDCALGKMGLSDAQAVLGAW-------------SSTPAF--ITADSAESWLWPLYNAG
HrcQEa        54 SHWRCALGAFSLANAAPLLNLL-------------SQCPLPAPGANPPDSDWQWALFNQY
HrcQXcv       11 CGFATHCRRISPNDAARLRLQLDAGQM--ELRI--AARDGLALLLNEDDDSLRVSIAGML
HrcQRsol          WQLHLARTPLAASAPLTLAIQSAQCRAELLIDGAHYPALHAIARETDRPRRLALGNLW

HrcQPss          LSAELANVLGALQPSASPEQQEEKFEYCELT--LQLSAGRLHSVLALPAHALADWLEQPL
HrcQEa       101 LSPELALLLGELQPDEPAQDGEVN---ARLH--VRLGDRHAECPLRFGHAQLAHWLSQPG
HrcQXcv       67 ----LADRLGAFAPLGLGAAEVIAFERDAEPDDCHGIGMTLGDLDAIALTASAPLLATLQ
HrcQRsol         ----LAPVLHALEDAGLGETQLTNLRRLK-ADAVHTSGPVLPLQIASAAHACRCDIHALD

HrcQPss          WNVNPP-----------LDHSGLALTFAMRLGRLSLPWQAIESLRPGDVLCPGEA--DFD
HrcQEa       156 WQSSRT-----------KLAGAITYSQPLVLGRITLCTEQLQALTAGDLLIPPVS--YFT
HrcQXcv            VGGLAPPAQQP----AWLAALRVNTRLRIGGRTASAALLQSLRPGDVLLHCTASAAV-
HrcQRsol         WHGVPPAPPAADPDTILHRFGALALPGRLRVASRRCRRGLLDTLAPGDILLGWNDATYRP

HrcQPss           AGHGALVMGPRHVRVRIVEHAERLQLEVL-QIEERTVTDRDELDQPISEAAETWAHDDA
HrcQEa       203 PDGQGSLTVAGQRLYGELQLP-HHFLLNHL---ESTAL----------------N------
HrcQXcv          ----TSGDVLWGIAGGVVLRAPVRLNLQQ-MILEASP-----------------------
HrcQRsol          ADEGTVHLAWGDARQPHLTATAHYKDGIVTTLDLHPL-----------------------

HrcQPss      263 QADDAYAQDAYGADA       DDDRYEDDAVEDEDGHEHHADPHPSSGPVSETPTGAFN
HrcQEa       238 --------------------SADDDALTEGSLPEYTGC----------------EDNPQLA
HrcQXcv          -------------------------TMQHDTFEPEV-----------------APSTSNVA
HrcQRsol         --------------------TDDDDAYPHDFAAPRTPA----------G-SSAGQGVPLE

HrcQPss          DLALPLTLRCGQINLTLGELATLVPGTVLEVPGIK-PGLAGLYYGERRLAQGELVDVEGR
HrcQEa       263 SLPLSLEVRCGRTALTLGELQRLQAGSVVTLDNVT-PGEAGLYHGDTLIARGELVDVEGH
HrcQXcv          ELELPVQLEVDQLALSLSTLSGLQPGQILELSVPVDQADIRLVVYGQTIGTGRLLAVGEH
HrcQRsol          LEVPVHLELAVMGMPLAELAALQPQHVLTLPVKIRDVSVRLVCHGQTLGHGQLVAVGEQ

HrcQPss          LGLQILQVDERG-----
HrcQEa       322 LGLQLTQLLLTSCQEVG
HrcQXcv          LGVQILSMSESTHADA-
HrcQRsol         LGLQIASIGKHHAER--
```

Figure S2 Alignments of HrcQ protein sequences from plant-pathogenic bacteria.
The following sequences were compared: HrcQ from *X. campestris* pv. *vesicatoria* (*Xcv*; GenBank accession number CAJ22054), HrcQ from *Ralstonia solanacearum* GMI1000 (*Rsol;* GenBank accession number CAD18012), HrcQ from *P. syringae* pv. *syringae* (*Pss;* GenBank accession number ACU65038) and HrcQ from *E. amylovora* (*Ea;* GenBank accession number AAB06004). Identical amino acids are shown in black boxes. Red letters represent amino acids which are encoded upstream of the annotated GTG start codon of *hrcQ* from *X. campestris* pv. *vesicatoria*.

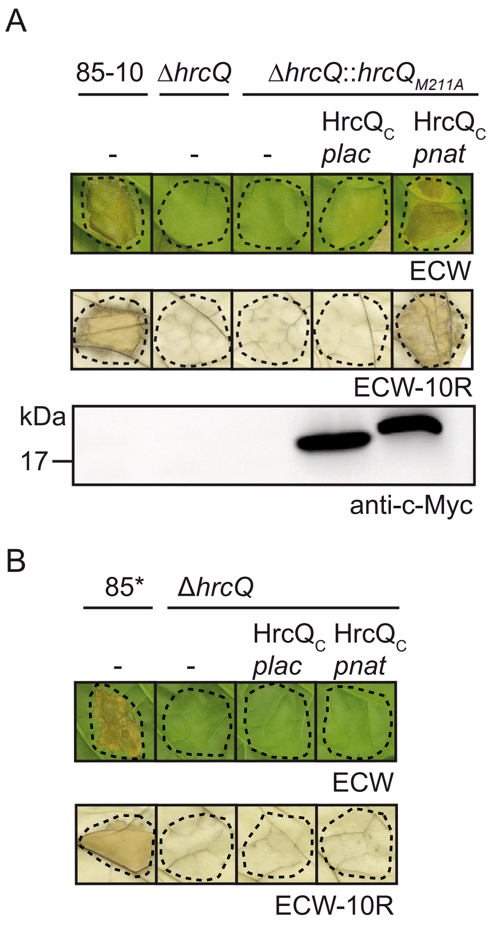

Figure S3 Complementation studies with HrcQ$_C$.
(A) *In trans* expression of *hrcQ$_C$* under control of the native promoter restores pathogenicity in a genomic *hrcQ$_{M211A}$* mutant. Strains 85-10, 85-10Δ*hrcQ* (Δ*hrcQ*) and 85-10Δ*hrcQ::hrcQ$_{M211A}$* (Δ*hrcQ::hrcQ$_{M211A}$*) without additional expression constructs (-) or containing expression constructs encoding HrcQ$_C$-c-Myc under control of the *lac* (*plac*) or the native (*pnat*) promoter were infiltrated into leaves of susceptible ECW and resistant ECW-10R pepper plants. Dashed lines indicate the infiltrated areas. Disease symptoms were photographed 8 dpi. For the better visualization of the HR, leaves were bleached in ethanol 2 dpi. For protein analysis, bacteria were grown in minimal medium and equal amounts of cell extracts were analysed by immunoblotting using a c-Myc epitope-specific antibody.
(B) HrcQ$_C$ does not complement the phenotype of a *hrcQ* deletion mutant. Strains 85* and 85*Δ*hrcQ* (Δ*hrcQ*) without expression constructs (-) or containing expression constructs encoding HrcQ$_C$-c-Myc under control of the *lac* (*plac*) or the native (*pnat*) promoter were infiltrated into leaves of susceptible ECW and resistant ECW-10R pepper plants. Plant reactions were documented as described in (A). Experiments were performed three times with similar results.

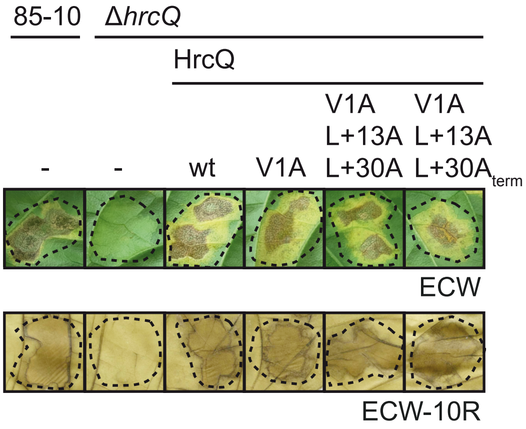

Figure S4 Ectopic expression of *hrcQ* derivatives with mutations in predicted start codons restores pathogenicity in an *X. campestris* pv. *vesicatoria hrcQ* deletion mutant strain.
Strains 85-10 and 85-10Δ*hrcQ* without plasmid (-) or containing expression constructs encoding HrcQ (wt) and mutant derivatives thereof with mutations in predicted start sites as indicated under control of the native promoter were infiltrated into leaves of susceptible ECW and resistant ECW-10R pepper plants. Dashed lines indicate the infiltrated areas. Disease symptoms were photographed 8 dpi. For the better visualization of the HR, leaves were bleached in ethanol 2 dpi. *term*, transcriptional terminator inserted upstream of the *hrcQ* promoter. Experiments were performed three times with similar results.

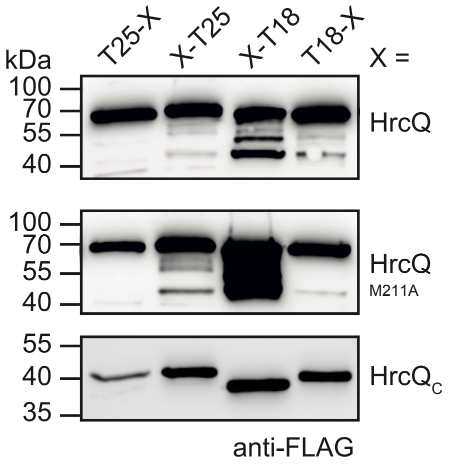

Figure S5 Synthesis of T25 and T18 fusions of HrcQ derivatives.
E. coli JM109 cells encoding T25 and T18 fusions as indicated under control of the *lac* promoter were grown in LB medium and gene expression was induced in the presence of IPTG. Protein extracts were analysed by immunoblotting using a FLAG epitope-specific antibody. Upper signals correspond to T18 and T25 fusion proteins, lower signals presumably represent degradation products.

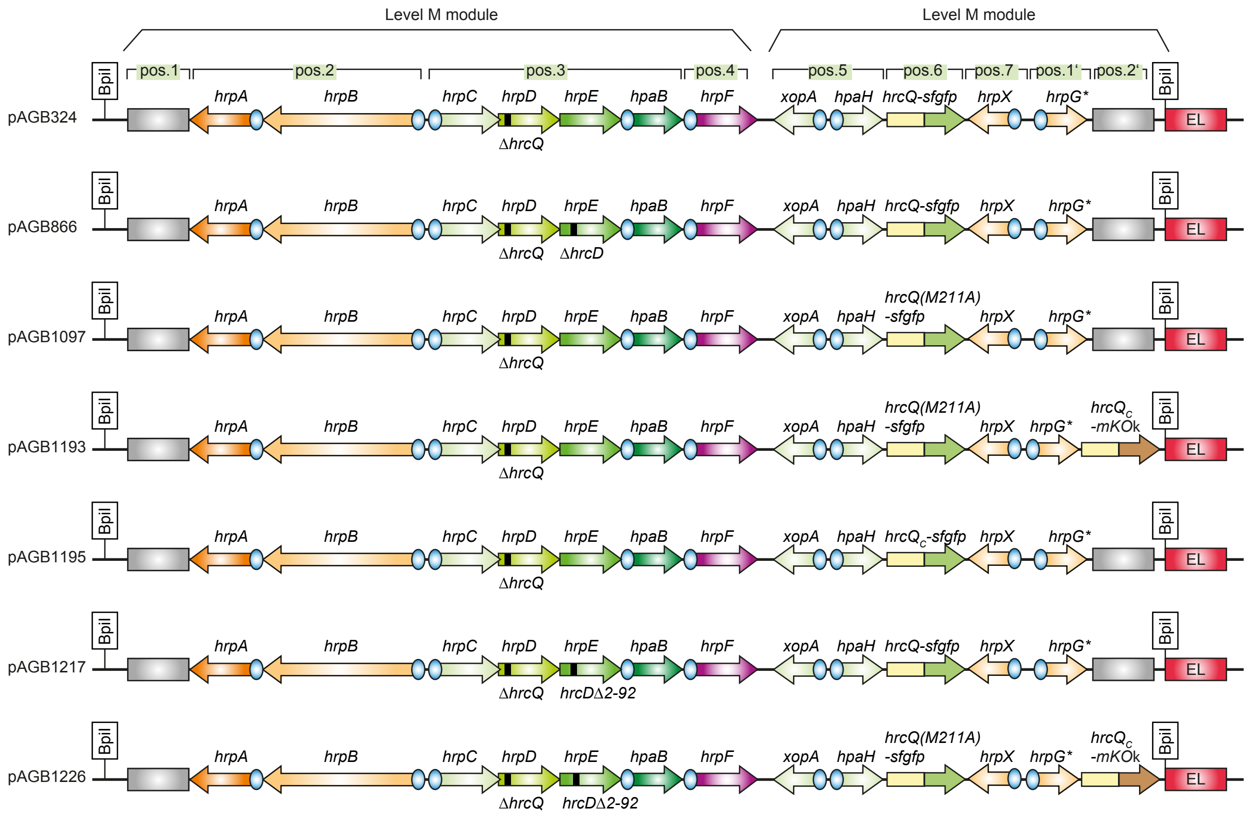

Figure S6 Schematic representation of modular T3S gene cluster constructs.
Genes are represented by arrows, promoters by blue circles. Grey rectangles represent dummy modules that can be replaced by reporter fusions. The deletions in *hrcQ* and *hrcD* are represented by black rectangles. The names of single operons and genes is given above the arrows. The constructs were assembled using the Golden Gate-based modular cloning technique as described previously (Hausner et al., 2019). Specific overhangs of gene or operon modules determine their positions (pos.) in the final level P construct which was assembled from two level M modules as indicated. Reporter genes encoding fluorescent fusions were inserted at positions 6 and 2' as shown. EL, end-linker.

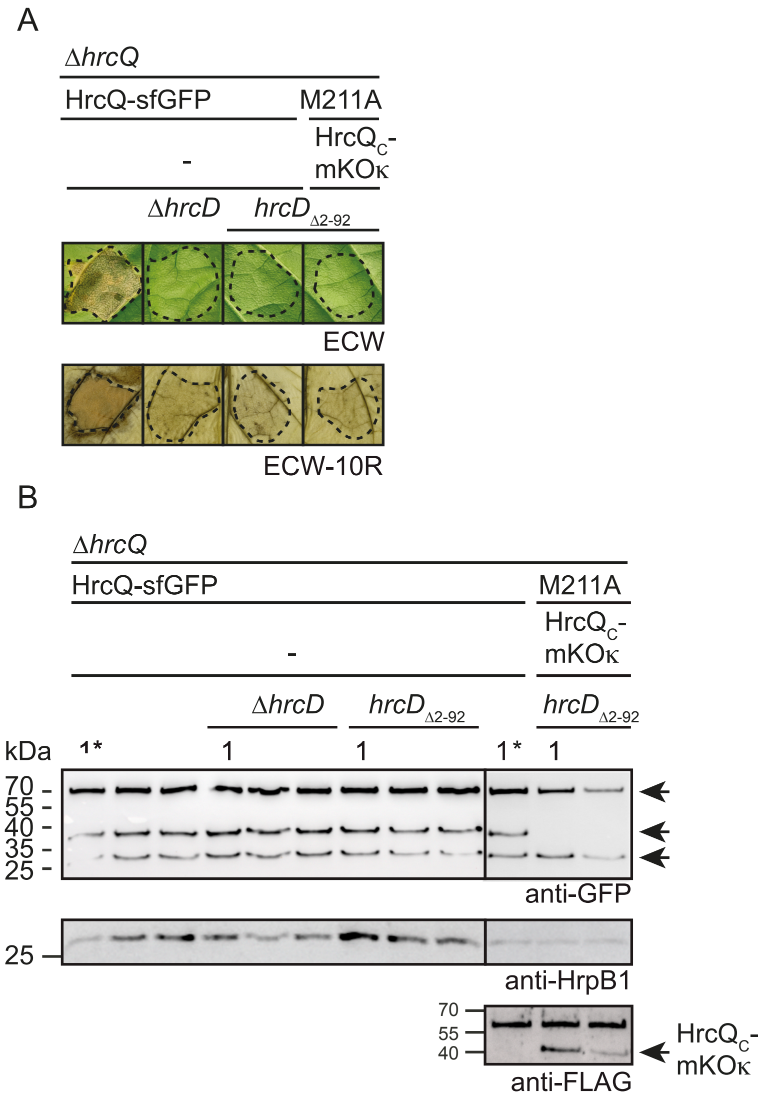

Figure S7 Analysis of HrcQ fusions in *hrcD* deletion mutant strains.
(A) Complementation studies with fluorescent HrcQ fusions. Strain 85*Δ*hrp*_fs*HAGX* with plasmids containing the modular *hrp-HAGX* construct (T3S gene cluster, accessory and regulatory genes) with deletions in *hrcQ* (Δ*hrcQ*), *hrcD* (Δ*hrcD*) or codons 2 – 92 of *hrcD* (Δ$hrcD_{2-92}$) and encoding HrcQ-sfGFP, $HrcQ_{M211A}$-sfGFP (M211A) or $HrcQ_C$-mKOκ as indicated was infiltrated into leaves of susceptible ECW and resistant ECW-10R pepper plants. Dashed lines indicate the infiltrated areas. Disease symptoms were photographed 8 dpi. For the better visualization of the HR, leaves were bleached in ethanol 2 dpi.
(B) Immunological detection of fluorescent HrcQ fusions. Three transconjugants (labeled 1, 2, and 3) of each strain described in (A) were cultivated in minimal medium (T3S-permissive conditions), and cell extracts were analysed by immunoblotting using antibodies specific for GFP and HrpB1. $HrcQ_C$-mKOκ was detected using a FLAG-specific antibody. The signals corresponding to the size of HrcQ-sfGFP, $HrcQ_C$-sfGFP and a GFP cleavage product are indicated by arrows in the upper blot. The arrow in the lower blot indicates the signal corresponding to the size of $HrcQ_C$-mKOκ. The upper signal results from unspecific binding of the antibody. The extract which is labeled with "1*" was loaded twice as indicated. Experiments were performed three times with similar results.
